# Systematic review and mixed treatment comparison of intravitreal aflibercept with other therapies for diabetic macular edema (DME)

**DOI:** 10.1186/s12886-015-0035-x

**Published:** 2015-05-15

**Authors:** Jean-Francois Korobelnik, Jos Kleijnen, Shona H Lang, Richard Birnie, Regina M Leadley, Kate Misso, Gill Worthy, Dominic Muston, Diana V Do

**Affiliations:** Université Bordeaux, ISPED, Centre INSERM U897-Epidemiologie-Biostatistique, F-33000 Bordeaux; Service d’Ophtalmologie, CHU de Bordeaux, Bordeaux, France; School for Public Health and Primary Care (CAPHRI), Maastricht University, Maastricht Limburg, the Netherlands; Kleijnen Systematic Reviews Ltd., Escrick Business Park, Escrick, York England, YO19 6FD UK; Global Health Economics and Outcomes Research, Bayer HealthCare Pharmaceuticals Inc., 100 Bayer Boulevard, Whippany New Jersey, 07981 USA; Truhlsen Eye Institute, University of Nebraska Medical Center, 985540 Nebraska Medical Center, Omaha, Nebraska 68198-5540 USA

**Keywords:** Intravitreal aflibercept, Diabetic macular edema (DME), Intravitreal ranibizumab, Meta-analysis, Systematic review

## Abstract

**Background:**

This was an indirect comparison of the effectiveness of intravitreal aflibercept (IVT-AFL) 2 mg every 8 weeks after 5 initial monthly doses (or if different periods, after an initial monthly dosing period) (2q8) and other diabetic macular edema (DME) therapies at doses licensed outside the USA.

**Methods:**

A comprehensive search was undertaken to source relevant studies. Feasibility networks were prepared to identify viable comparisons of 12-month outcomes between IVT-AFL 2q8 and therapies licensed outside the USA, which were assessed for clinical and statistical homogeneity. Pooled effect sizes (mean difference [MD] and relative risk/risk ratio [RR]) were calculated using fixed- and random-effects models. Indirect comparisons were performed using Bucher analysis. If at least one ‘head-to-head’ study was found then a mixed treatment comparison (MTC) was performed using Bayesian methods. Two 12-month comparisons could be undertaken based on indirect analyses: IVT-AFL 2q8 versus intravitreal ranibizumab (IVR) 0.5 mg as needed (PRN) (10 studies) and IVT-AFL 2q8 versus dexamethasone 0.7 mg implants (three studies).

**Results:**

There was an increase in mean best-corrected visual acuity (BCVA) with IVT-AFL 2q8 over IVR 0.5 mg PRN by 4.67 letters [95% credible interval (CrI): 2.45–6.87] in the fixed-effect MTC model (10 studies) and by 4.82 letters [95% confidence interval (CI): 2.52–7.11] in the Bucher indirect analysis (four studies). IVT-AFL 2q8 doubled the proportion of patients gaining ≥ 10 Early Treatment Diabetic Retinopathy Study letters at 12 months compared with dexamethasone 0.7 mg implants (RR = 2.10 [95% CI: 1.29–3.40]) in the fixed-effect model. There were no significant differences in safety outcomes between IVT-AFL 2q8 and IVR 0.5 mg PRN or dexamethasone 0.7 mg implants.

**Conclusions:**

Studies of IVT-AFL 2q8 showed improved 12-month visual acuity measures compared with studies of IVR 0.5 mg PRN and dexamethasone 0.7 mg implants based on indirect comparisons. These analyses are subject to a number of limitations which are inherent in indirect data comparisons.

**Electronic supplementary material:**

The online version of this article (doi:10.1186/s12886-015-0035-x) contains supplementary material, which is available to authorized users.

## Background

Severe retinopathy and presence of diabetic macular edema (DME) are associated with vision loss in patients with diabetes [1]. Although focal laser photocoagulation has been the standard of care for DME [2] it can only slow progression and its ability to reverse vision loss is low [3]. Awareness of the role of vascular endothelial growth factors (VEGF and placental growth factor [PIGF]) and inflammatory mediators in stimulating retinal vasculogenesis and angiogenesis [4] has led to the development and widespread use of anti-VEGF agents that can target these pathways [5,6].

Intravitreal aflibercept (IVT-AFL), which is composed of extracellular domains from human VEGF receptors 1 and 2 fused to the Fc portion of human immunoglobulin-G1 (IgG1), is a VEGF-A and PIGF inhibitor that blocks retinal cell migration and proliferation. Preclinical studies have shown that it has a longer duration of action than other anti-VEGF agents, and has 100-fold greater binding affinity to VEGF-A than intravitreal ranibizumab (IVR) (a recombinant humanized monoclonal antibody that inhibits VEGF-A) [7-10]. Clinical studies have demonstrated the efficacy and safety of these anti-VEGF agents compared with laser in DME patients [11-16]. The IVT-AFL studies have supported its European license (i.e., five 2 mg injections every 4 weeks followed by 2 mg injections every 8 weeks [2q8]; with no requirement for monitoring between injections; after the first 12 months of treatment with IVT-AFL, the treatment interval may be extended based on visual and anatomic outcomes; the schedule for monitoring should be determined by the treating physician).

Meta-analyses have been undertaken to compare anti-VEGF agents, based on a lack of direct comparisons prior to the recent publication of the Protocol T study [17-20]. However, some analyses have pooled IVR studies regardless of the posology or the nature of the comparator, and comparisons involving IVT-AFL have been based on only the DA VINCI study, which differs in design from the more recent phase III VIVID-DME and VISTA-DME studies in many aspects, including loading phase (DA VINCI included three initial loading doses in some arms compared with five in VIVID-DME and VISTA-DME) [11,13]. In addition, the meta-analysis by Virgili et al. [18] contained a limited and exploratory indirect comparison of differences in efficacy among anti-VEGF agents (3-line gains only).

The aims of this study were to systematically identify and review studies informing the clinical effectiveness of IVT-AFL 2q8 in relation to comparator treatments and regimens licensed outside of the USA for the management of DME through mixed treatment and indirect comparison methods. The comparators of interest were: IVR 0.5 mg as needed (PRN), and implants of dexamethasone 0.7 mg or fluocinolone acetate 0.2 μg/day. Unlike the meta-analysis by Virgili et al. [18], this study will consider a broader range of outcomes (including reporting of best-corrected visual acuity [BCVA] based on letters, which is used in most studies, rather than logarithm of the minimal angle of resolution) and will focus on a comparison of licensed anti-VEGF agents. The need for such an approach was supported by the limited outcome of the Virgili et al. meta-analysis [18].

## Methods

### Search strategy

A comprehensive search was undertaken to identify relevant studies. To reduce the risk of bias and error, the database selection, systematic literature search and review adhered to guidelines for the Institut fur Qualitat und Wirtschaftlichkeit im Gesundheitswesen (IQWiG) methods guide (Version 4.0), the Cochrane Collaboration and Centre for Review and Dissemination (York, UK) [21-23].

Search strategies were developed specifically for each database and used a variety of synonyms for DME. The following databases were searched from inception: Medline (1946–2013/10); Medline In-Process Citations and Daily Update (up to 2013/10/13); Embase (1974–2013/10); Cochrane Central Register of Controlled Trials (up to 2013/10/15). The main search strategy for Embase is listed in Additional file 1: Appendix 1. A number of other searches were also undertaken, including other databases (rapid appraisal), websites, and congress abstracts, which are listed in Additional file 1: Appendix 2. The bibliographies of identified research and review articles were also checked for studies. In addition, the final included papers were checked on PubMed for retractions and errata. Additional data (including abstracts for any unpublished studies at the time of literature review) were provided by Bayer HealthCare (Berlin, Germany).

### Inclusion criteria

Studies were included if they met the PICOS criteria (populations, interventions, comparators, outcomes and study design) and prespecified requirements for inclusion in indirect and mixed treatment analyses (Table 1). The additional criteria for study selection exclude studies that cannot inform mixed treatment comparisons of IVT-AFL 2q8 versus comparators of interest (IVR 0.5 mg PRN, and implants of dexamethasone 0.7 mg or fluocinolone acetonide 0.2 μg/day) for outcomes at 12 months. The population criterion (‘patients with DME’) was deliberately inclusive, irrespective at this stage of features such as central involvement, prior treatments used or baseline visual acuity. This systematic review included studies that were compliant with the Declaration of Helsinki, had protocols approved by relevant country- and study-specific Institutional Review Boards/independent ethics committees, and enrolled patients that provided informed consent to participate in them.

**Table 1:** **An overview of the PICOS and other criteria used for study inclusion and exclusion**

**Criteria**	**Inclusion**	**Exclusion**
Study design	Published and unpublished randomized controlled studies	Systematic or non-systematic reviews and meta-analyses
	Dose or frequency comparison studies	Preclinical studies, retrospective prognostic studies, and case reports
	Ad-hoc analyses of randomized controlled study data	Editorials, commentaries, letters, and consensus reports
	Crossover randomized controlled studies	Pilot studies (if phase not mentioned), phase I and II randomized controlled studies (to be included as second-level evidence, if primary evidence is unavailable)
		Controlled observational studies (to be included as second-level evidence, if primary evidence is unavailable)
		Separate searches will be performed as required
		Single dose of intervention studies
		Studies of less than 3 months follow-up
Population	Patients with DME	
Interventions	Eylea/VEGF Trap-Eye/aflibercept	Systemic treatments (alone or in combination with intervention)
	Anti-VEGF treatments (any including ranibizumab/Lucentis, bevacizumab/Avastin, and pegaptanib/Macugen)	Surgery (alone or in combination with intervention)
		Subtenon injections
	Intravitreal steroids (any including triamcinolone, fluocinolone acetonide/Iluvien, dexamethasone/Ozurdex, and implants)	
	Laser treatments	
	NOTE the intervention should be to treat the DME not to treat cataracts	
	The above interventions can be included if combined with other treatments (e.g., eye drops) except the exclusions	
Comparators	Placebo, best standard care, masked control, sham, and eye drops	Systemic treatments (alone or in combination with intervention)
	Any intervention (from those listed as interventions)	Surgery (alone or in combination with intervention)
	NOTE: this can be a single treatment/implant	
Clinical Outcomes	Number of injections/visits/assessments	
	BCVA (mean change from baseline, mean average change from baseline, as measured by ETDRS score or Snellen equivalent)	
	Loss of ≤ 15, ≥ 15, ≥ 30 ETDRS letters	
	Gain of ≥ 0, 10, 15, 30 ETDRS letters	
	20/40 vision or better (Snellen chart)	
	20/200 or worse (Snellen chart)	
	Reduction in laser use	
	Anatomical changes (e.g., change in CNV and lesion area, central foveal thickness, and fluid on OCT)	
	Health-related quality of life (EQ-5D, NEI VFQ-25, and other scales)	
	Treatment discontinuations	
	Serious AE (all serious AE, all ocular serious AE, death, endophthalmitis, uveitis, retinal tear, diabetic macular/retinal edema, reduced visual acuity, vitreous hemorrhage, corneal abrasion, and any others)	
	AE (all AE, all ocular AE, all non-ocular AE, retinal detachment, retinal ischemia, lens damage, all grades of ocular inflammation, eye pain, increased ocular pressure, retinal degradation, macular edema, cataract, neovascularization, and any others)	
	Serious non-ocular AE (all, non-fatal cardiac infarction, non-fatal stroke, non-ocular hemorrhage, hypertension, serious systemic events, arterial thrombotic events, and venous thrombotic events)	
Language	Any	
Additional criteria necessary for inclusion in indirect and mixed treatment analysis		Studies that were connected by one arm only and did not form a closed network, unless they included comparators of interest
		Studies that formed loops but did not lie along the path between IVT-AFL 2q8 versus comparators of interest (IVR 0.5 mg PRN, or implants of dexamethasone 0.7 mg or fluocinolone acetonide 0.2 μg/day)
		Studies that did not report 12-month outcomes

AE, adverse event; BCVA, best-corrected visual acuity; CNV, choroidal neovascularization; DME, diabetic macular edema; EQ-5D, EuroQoL-5D; ETDRS, Early Treatment Diabetic Retinopathy Study; IVR, intravitreal ranibizumab; IVT-AFL, intravitreal aflibercept; NEI VFQ-25, National Eye Institute 25-item Visual Function questionnaire; OCT, optical coherence tomography; PRN, as-needed; VEGF, vascular endothelial growth factor.

### Data extraction and quality assessment

Titles and abstracts identified through the search strategies described were independently screened by two reviewers, and any references which did not meet the inclusion criteria listed previously were excluded. During the screening of conference abstracts, only studies which specifically mentioned randomization and which reported extractable outcome data (or baseline or subgroup data) were included. Full paper copies were obtained for the remaining references, which were examined in detail to determine whether they met the inclusion criteria. All papers excluded at this second stage of the screening process were documented along with the reasons for exclusion. Any discrepancies between reviewers were resolved through discussion or the intervention of a third reviewer. A similar approach was undertaken for data extraction and quality assessment.

Data extraction forms were designed and piloted by reviewers. To avoid duplication of data where studies (or study populations) had multiple publications, the most recent and complete report was used as the main reference, but additional details were extracted from the other publications as necessary.

The quality of each individual study was assessed to identify any potential sources of bias using the Cochrane Risk of Bias Tool for randomized controlled trials [22]. In brief, bias was graded as low risk, high risk or unclear in several domains (selection, performance, detection, attrition, reporting, and other).

### Statistical analyses

The analysis approach was predefined in the study protocol. Based on the descriptive summary of all of the included studies, a feasibility assessment was undertaken to determine which comparisons and outcomes could be included. Studies could not be included in indirect analyses if: they were connected by one arm only and did not form a closed network, unless they included comparators of interest; formed loops but did not lie along the path between IVT-AFL 2q8 and comparators of interest (IVR 0.5 mg PRN, and implants of dexamethasone 0.7 mg or fluocinolone acetonide 0.2 μg/day); or did not report 12-month outcomes. For any direct ‘head-to-head’ comparisons between two treatments, studies were pooled using meta-analysis, following methods recommended by the Cochrane Handbook [22]. Forest plots of effect sizes were prepared for each of the outcomes. Dichotomous outcomes were reported as relative risks/risk ratios (RR) and odds ratios (OR) with 95% confidence intervals (CI) and continuous outcomes were reported as mean differences (MD) with 95% CI.

Both clinical and statistical homogeneity were addressed in accordance with published methods [24,25]. Statistical homogeneity was assessed for the direct comparisons with the I^2^ statistic [24]. Studies were only considered to be sufficiently similar and suitable for meta-analysis if I^2^ < 75% based on the following categorization of heterogeneity: low (0–25%), moderate (26–75%) and high (> 75%) [24]. The judgment of clinical homogeneity was based on study design, risk of bias, inclusion/exclusion criteria, baseline participant characteristics and treatment regimen.

Data were pooled where studies were considered to be clinically and statistically homogeneous, and pooled effect sizes (RR, OR, MD) and 95% CIs were calculated using both fixed-effect and random-effects models using inverse variance or Mantel-Haenszel methods. If there was a connected network of three or more studies, then indirect treatment comparisons and mixed treatment comparisons (MTC) were performed. The underlying assumptions of homogeneity, similarity and consistency in the network were evaluated, as reported in Song et al. [25]. All indirect comparisons and MTC methods followed the guidance of the International Society for Pharmacoeconomic and Outcomes Research (ISPOR) taskforce recommendations for the conduct of indirect and MTC meta-analysis [26]. Indirect comparisons were performed according to the method developed by Bucher et al. [27]. Where feasible, an indirect estimate of the effect size was calculated from the results of the corresponding direct meta-analyses. If at least one ‘head-to-head’ study was found, then an MTC (using a network of both ‘head-to-head’ and indirect comparisons) was performed using Bayesian methods. MDs, RRs and ORs (with 95% credible interval [CrI]) were calculated for each outcome and available treatment comparison using both fixed- and random-effects models. Model fit was assessed and compared between fixed- and random-effects models using the deviance information criterion (DIC) [28]. MTC analyses were performed using WinBUGS version 1.4.3 and the direct meta-analyses were performed using Cochrane Review Manager Version 5.2 (RevMan 5.2). Sensitivity analysis was used to investigate any studies which might not fulfill the assumptions of similarity or homogeneity.

## Results

### Feasibility assessments

**Figure 1:**
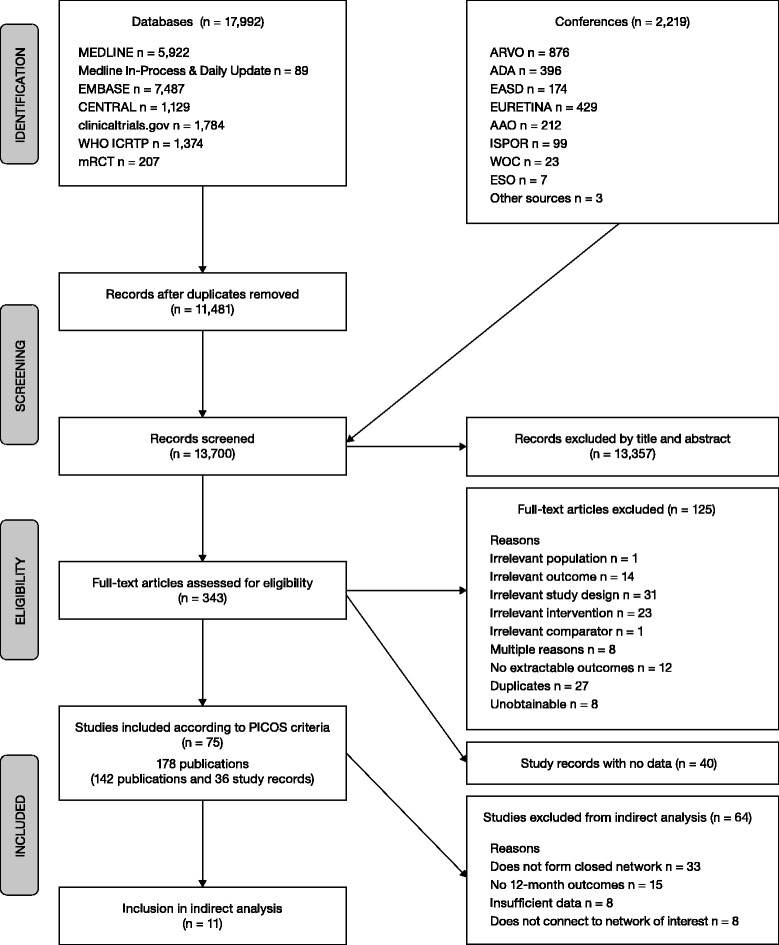
Flow chart of the literature search.

**Figure 2:**
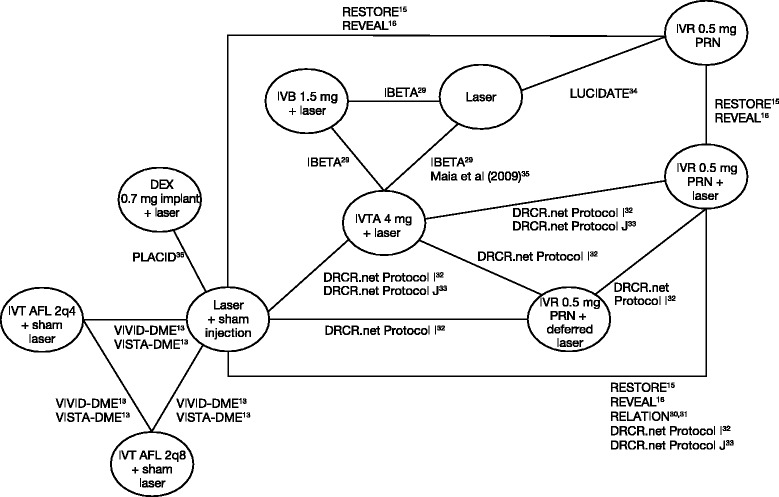
Final feasibility network at 12 months, showing direct comparisons by drug, comparator and dose. IVB, intravitreal bevacizumab; IVR, intravitreal ranibizumab; IVT-AFL, intravitreal aflibercept; IVTA, intravitreal triamcinolone acetonide.

A flow chart illustrating the results of the search strategy is shown in Figure 1. The systematic review identified 75 studies that satisfied the PICOS criteria. These studies are summarized in Additional file 1: Appendix 3. Of these, 11 studies could be included since they provided data that could inform the indirect analyses of interest (Figure 2) [13,15,16,29-36]. The RISE/RIDE [37] studies did not inform the indirect analyses because they do not provide evidence of IVR 0.5 mg PRN, dexamethasone 0.7 mg implants or IVT-AFL 2q8, and did not lie along the path between IVT-AFL 2q8 and comparators of interest. No network could be formed to provide a comparative assessment with fluocinolone acetate, so this indirect comparison was also not possible. Data for two studies were based on an abstract and unpublished clinical study reports at the time of review; however, these studies are now published in full [13].

Two analyses were performed. Firstly, IVT-AFL 2q8 versus IVR 0.5 mg PRN using indirect analyses (Bucher and MTC) based on the defined efficacy outcomes (mean change from baseline in BCVA based on Early Treatment Diabetic Retinopathy Study [ETDRS] score; gain of ≥ 10 or ≥ 15 letters; and loss of ≥ 10 or ≥ 15 letters) and safety outcomes (all adverse events [AEs]; all serious AEs; all ocular AEs; all serious ocular AEs; all non-ocular AEs; all serious non-ocular AEs; eye pain; cataract; hypertension and all causes of mortality). Mean change in BCVA was also adjusted for baseline visual acuity score by including a treatment interaction effect common across interventions in the MTC model [38]. Secondly, IVT-AFL 2q8 versus dexamethasone 0.7 mg implants using an indirect analysis (Bucher) with defined efficacy (gain ≥ 10 letters) and safety (macular edema, reduced visual acuity, vitreous hemorrhage, eye pain, increased intraocular pressure, and cataract) outcomes.

### Efficacy outcomes: IVT-AFL 2q8 versus IVR 0.5 mg PRN

The assessment of clinical similarity showed that all studies were randomized, and the majority had similar designs (i.e., multicenter, double-blinded). With respect to the inclusion/exclusion criteria, patients with macular edema were classified by a range of anatomical and functional measures. The main inclusion criteria included significant DME [29], DME [30,31], focal or diffuse DME [15,16,36], DME secondary to diabetes involving the center of the macula [13], retinal thickening due to DME [32-34] or clinically significant macula edema in patients with diabetic retinopathy [35]. The visual acuity at baseline is summarized in Table 2; studies reported that visual acuity had to be 20/40 or worse [13,29] or 20/32 or worse [15,16], or patients had to have a BCVA letter score of 34–70 [36], 39–78 at 4 meters [15,16], 24–73/78 [13,32,33] or 55–79 at 1 meter [34]. It was difficult to compare baseline characteristics, due to lack of consistency in reporting these items and absence of these data, particularly in studies published only in abstract form. Three studies were most dissimilar in this regard [29,34,35].

**Table 2:** **An overview of the studies (n = 11) included in the final analyses**

**Reference**	**Phase**	**Design**	**Randomized patients (n)**	**Inclusion**	**Interventions**	**Baseline ETDRS score, mean (SD)**	**Follow-up (months)**	**Primary outcome**	**Mean change in BCVA (letters) at Month 12**
VIVID-DME [13]	III	Randomized, double-blind, multicenter	136 135 135	Patients with DME secondary to diabetes mellitus. BCVA ETDRS letter score between 24 and 73 in the study eye	IVT-AFL 2q4* IVT-AFL 2q8* Laser*	60.8 (10.7) 58.8 (11.2) 60.8 (10.6)	12	Mean change in BCVA (ETDRS letters score) at Week 52	+10.5 +10.7 +1.2
VISTA-DME [13]	III	Randomized, double-blind, multicenter	156 154 156	Patients with DME secondary to diabetes mellitus. BCVA ETDRS letter score between 24 and 73 in the study eye	IIVT-AFL 2q4* IVT-AFL 2q8* Laser*	58.9 (10.8) 59.4 (10.9) 59.7 (11.0)	12	Mean change in BCVA (ETDRS letters score) at Week 52	+12.5 +10.7 +0.2
IBETA [29] Abstract	III	Randomized, open, single center	23 21 20	Clinically significant DME. Snellen logarithm of minimum angle of 20/40 or worse	Laser fixed → PRN IVB 1.5 mg + laser IVTA 4 mg + laser	NR NR NR	12	Outcomes included BCVA, OCT-CMT at Week 52	+9.5 +11.5 +12.5
RESTORE [15]	III	Randomized, double-blind, multicenter	111 116 118	Focal or diffuse DME. BCVA letter score between 39 and 78	Laser fixed q4 → PRN* IVR 0.5 mg q4 → PRN* IVR 0.5 mg + laser	62.4 (11.1) 64.8 (10.1) 63.4 (10.0)	12	Mean average change in BCVA from baseline to Month 1 through 12	+0.8 +6.1 +5.9
REVEAL [16] Abstract	III	Randomized, double-blind, multicenter	133 132 131	Focal or diffuse DME. BCVA letter score between 39 and 78	IVR 0.5 mg q4 → PRN* IVR 0.5 mg + laser Laser fixed q4 → PRN*	NR NR NR	12	Mean average change in BCVA from baseline to Month 1 through 12	+6.6 +6.4 +1.8
RELATION [30,31] Abstracts	III	Randomized, double-blind, multicenter	85 43	DME	IVR 0.5 mg + prompt laser Laser fixed q4 → PRN*	NR NR	12	Changes in BCVA, OCT-CRT, and FA	+6.5 +1.4
DRCR.net Protocol I [32]	III	Randomized, double-blind, multicenter	293 187 188 186	DME. BCVA letter score between 24 and 78	Laser fixed q4 → PRN* IVR 0.5 mg + prompt laser IVR 0.5 mg + deferred laser IVTA 4 mg + laser	NR NR NR NR	12 (maximum 36)	Mean change in BCVA at month 12	+3 +9 +9 +4
DRCR.net Protocol J [33]	III	Randomized, double-blind, multicenter	123 113 109	DME and presence of severe NPDR or PDR. ETDRS letter score ≥ 24	Laser fixed* IVR 0.5 mg + laser IVTA 4 mg + laser	NR NR NR	12	Mean change in visual acuity from baseline to Week 14	−6 −4 −5
LUCIDATE [34] Abstract	IV	Randomized, open, single center	11 11	DME. BCVA letter score between 55 and 79	IVR 0.5 mg q4 → PRN Laser fixed → PRN	NR NR	11	BCVA ETDRS VA, FA, OCT, microperimetry, full-field and multifocal ERG at Week 48	+6.0 −0.9
Maia et al. (2009) [35]	II/III	Randomized, single-blind, single center	22 22	DR and CSME. ETDRS severity level 65	Laser fixed → PRN IVTA 4 mg + laser	NR NR	12	Changes in BCVA, CMT, and TMV	+3** +16**
PLACID [36]	II	Randomized, double-blind, multicenter	126 127	Diffuse DME. BCVA letter score between ≥ 34 and ≤ 70	Dexamethasone fixed → PRN Laser fixed → PRN*	57 (9.4) 57.5 (9.5)	12	Proportion who gained ≥ 10 letters from baseline to Month 12	NA

BCVA, best-corrected visual acuity; CMT, central macular thickness; CRT, central retinal thickness; CSME, clinically significant macular edema; DME, diabetic macular edema; DR, diabetic retinopathy; ERG, electroretinography; ETDRS, Early Treatment Diabetic Retinopathy Study; FA, fluorescein angiography; IVB, intravitreal bevacizumab; IVR, intravitreal ranibizumab; IVT-AFL, intravitreal aflibercept; IVTA, intravitreal triamcinolone acetonide; NA, not available; NPDR, non-proliferative diabetic retinopathy; NR, not reported; OCT, optical coherence tomography; PDR, proliferative diabetic retinopathy; PRN, as needed; TMV, total macular volume; VA, visual acuity.

*Includes sham. **Published as logarithm of the minimum angle of resolution used, converted here to ETDRS letters using Gregori NZ, et al. Retina. 2010; 30:1046-50.

Treatment interventions are listed in Table 2, and treatment regimens are described in detail in Additional file 1: Appendix 4. Most studies employed a laser control arm (as described in Table 2). The need for additional treatment or retreatment was assessed on an as-needed basis in studies with an active as-needed treatment arm, and on a fixed-term basis in studies with a fixed-dose active treatment arm; control arms (which were largely laser) were suitable for retreatment after an appropriate timeframe. In the VIVID-DME and VISTA-DME studies, additional/rescue treatment with laser was permitted in the IVT-AFL groups, and anti-VEGF treatment was permitted in the laser arms [13]. The decision for retreatment with injections was guided by optical coherence tomography (OCT) or vision stability in most studies and retreatment with laser was usually guided by ETDRS guidelines. The studies varied regarding the risk of bias. Six studies [13,15,29,33,35] all had a high risk of bias for at least one domain, but four studies [16,30-32,34] did not have high risk of bias in any domain (Additional file 1: Appendix 5). Based on clinical assessments, the 10 studies included (particularly the four studies included in the Bucher analysis) were considered sufficiently similar for fixed-effect analysis.

#### Direct and indirect analyses

**Figure 3:**
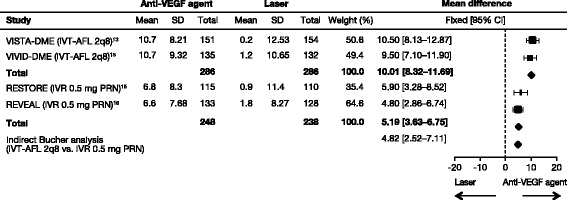
Direct comparison of IVT-AFL 2q8 (plus sham laser) or IVR 0.5 mg PRN (plus sham laser) versus laser (plus sham injection) for mean BCVA change from baseline in key studies. Indirect comparison (IVT-AFL 2q8 vs IVR 0.5 mg PRN) (Bucher analysis) also shown. BCVA, best-corrected visual acuity; CI, confidence interval; IVR, intravitreal ranibizumab; IVT-AFL, intravitreal aflibercept; PRN, as-needed; VEGF, vascular endothelial growth factor.

IVT-AFL 2q8 and IVR 0.5 mg PRN could be directly compared via a common comparator of laser (plus sham injection) in four studies (Figure 3) [13,15,16]. These comparisons showed that treatment with IVT-AFL 2q8 resulted in a significantly greater improvement in BCVA mean change from baseline compared with laser (MD = 10.01 [95% CI: 8.32–11.69]). IVR 0.5 mg PRN also showed a significant improvement compared with laser (MD = 5.19 [95% CI: 3.63–6.75]). Only two studies were included in each comparison but their results were statistically homogeneous (I^2^ = 0%). Results of additional direct comparisons are summarized in Additional file 1: Appendix 6.

The results from the indirect and MTC analyses showed that IVT-AFL 2q8 improved the mean BCVA change from baseline to a greater extent than IVR 0.5 mg PRN. The MD estimates from the fixed-effect models were 4.67 [95% CrI: 2.45–6.87] (MTC; 10 studies) (Table 3A) and 4.82 [95% CI: 2.52–7.11] (Bucher; four studies) (Table 3B). This effect remained after adjustment for baseline visual acuity score (the MD estimate from the MTC fixed-effect model was 4.12 [95% Crl: 1.47–6.81]) (Additional file 1: Appendix 7). IVT-AFL 2q8 significantly reduced the loss of ≥ 10 ETDRS letters at 12 months using MTC (RR = 0.27 [95% CrI: 0.07–0.90]) (six studies) but not Bucher analysis (RR = 0.31 [95% CI: 0.09–1.04]) (four studies) (Table 3). This effect remained after adjustment for baseline visual acuity score using MTC (RR = 0.11 [95% CrI: 0.02–0.46]) (Additional file 1: Appendix 7). There was no significant difference between IVT-AFL 2q8 and IVR 0.5 mg PRN treatments for gain of ≥ 10 ETDRS letters, gain of ≥ 15 ETDRS letters or loss of ≥ 15 ETDRS letters in either MTC or Bucher analyses, with or without adjustment for baseline visual acuity (Table 3, Additional file 1: Appendix 7). One study included in the analysis was conducted in a regional population group (China, Hong Kong, Japan, South Korea, Singapore and Taiwan) raising a question about the effects of an Asian subpopulation effect on the main analysis. When this study (Ohji et al. [16]) was excluded, the overall effect remained similar; the difference in mean BCVA change from baseline was 4.11 [95% CI: 0.99–7.22] by Bucher analysis.

**Table 3:** **Indirect comparisons of the effects of IVT-AFL 2q8 versus IVR 0.5 mg PRN on 12-month visual outcomes using (A) MTC and (B) Bucher analyses**

**(A)**			
**MTC**	**Studies (n)**	**FE: effect size [95% CrI]**	**RE: effect size [95% CrI]**
BCVA mean change from baseline	10 studies (n = 3060)*	MD = 4.67 [2.45–6.87]	MD = 4.67 [1.85–7.52]
Gain ≥ 10 ETDRS letters	6 studies (n = 2810)**	RR = 1.32 [0.98–1.78]	RR = 1.19 [0.90–1.57]
		OR = 1.64 [0.97–2.78]	OR = 1.59 [0.75–3.35]
Loss ≥ 10 ETDRS letters	6 studies (n = 2810)**	RR = 0.27 [0.07–0.90]	RR = 0.28 [0.06–1.29]
		OR = 0.27 [0.07–0.90]	OR = 0.26 [0.05–1.31]
Gain ≥ 15 ETDRS letters	6 studies (n = 2810)**	RR = 1.78 [0.96–3.29]	RR = 1.42 [0.93–2.24]
		OR = 1.90 [0.95–3.75]	OR = 1.87 [0.87–4.16]
Loss ≥ 15 ETDRS letters	6 studies (n = 2810)**	RR = 0.13 [0.004–1.35]	RR = 0.14 [0.007–1.52]
		OR = 0.13 [0.004–1.35]	OR = 0.14 [0.006–1.53]
**(B)**			
**Bucher**	**Studies (n)**	**FE: effect size [95% CI]**	**RE: effect size [95% CI]**
BCVA mean change from baseline	4 studies (n = 1611)***	MD = 4.82 [2.52–7.11]	MD = 4.82 [2.52–7.11]
Gain ≥ 10 ETDRS letters	4 studies (n = 1611)***	RR = 0.993 [0.65–1.52]	RR = 1.00 [0.60–1.66]
		OR = 1.32 [0.74–2.35]	OR = 1.32 [0.65–2.68]
Loss ≥ 10 ETDRS letters	4 studies (n = 1611)***	RR = 0.31 [0.09–1.04]	RR = 0.31 [0.09–1.09]
		OR = 0.28 [0.08–0.99]	OR = 0.27 [0.08–0.94]
Gain ≥ 15 ETDRS letters	4 studies (n = 1611)***	RR = 1.49 [0.78–2.85]	RR = 1.49 [0.78–2.85]
		OR = 1.74 [0.83–3.65]	OR = 1.74 [0.83–3.65]
Loss ≥ 15 ETDRS letters	4 studies (n = 1611)***	RR = 0.24 [0.03–1.90]	RR = 0.26 [0.03–2.11]
		OR = 0.23 [0.03–1.86]	OR = 0.23 [0.03–1.86]

*VIVID-DME, VISTA-DME, IBETA, RESTORE, REVEAL, RELATION, DRCR.net Protocol I, DRCR.net Protocol J, LUCIDATE, and Maia et al. [13,15,16,29-35].

**VIVID-DME, VISTA-DME, RESTORE, REVEAL, DRCR.net Protocol I, and DRCR.net Protocol J [13,15,16,32,33].

***VIVID-DME, VISTA-DME, RESTORE, and REVEAL [13,15,16].

BCVA, best-corrected visual acuity; CI, confidence interval; CrI, credible interval; ETDRS, Early Treatment Diabetic Retinopathy Study; FE, fixed effects; IVR, intravitreal ranibizumab; IVT-AFL, intravitreal aflibercept; MD, mean difference; MTC, mixed treatment comparison; OR, odds ratio; PRN, as needed; RE, random effects; RR, relative risk/risk ratio.

### Efficacy outcomes: IVT-AFL 2q8 versus dexamethasone 0.7 mg implants

Two studies [13] reported data to allow a direct analysis between IVT-AFL 2q8 (plus sham laser) versus laser (plus sham injection) for the outcome ‘gain of ≥ 10 ETDRS letters’, with an RR = 2.50 [95% CI: 1.97–3.17]. This analysis showed moderate heterogeneity (I^2^ = 56%). Only one study [36] reported a gain of ≥ 10 ETDRS letters for the comparison of dexamethasone 0.7 mg (plus laser) versus laser (plus sham implant); therefore, no direct meta-analysis was possible. This study reported an RR = 1.18 [95% CI: 0.77–1.79]. Indirect analyses of these three studies showed that IVT-AFL 2q8 improved the proportion of patients gaining ≥ 10 ETDRS letters at 12 months compared with dexamethasone 0.7 mg implants (RR = 2.10 [95% CI: 1.29–3.40], fixed-effect model) (Table 4). Analyses of other efficacy outcomes were not feasible.

**Table 4:** **Indirect comparison (Bucher analysis) of the effects of IVT-AFL 2q8 versus dexamethasone 0.7 mg implants on 12-month visual outcomes**

**Outcome**	**Studies (n)**	**FE: effect size [95% CI]**	**RE: effect size [95% CI]**
Gain ≥ 10 ETDRS letters	3 studies (n = 1123)*	RR = 2.10 [1.29–3.40]	RR = 2.10 [1.21–3.66]
		OR = 3.51 [1.79–6.88]	OR = 3.52 [1.60–7.72]

*VIVID-DME, VISTA-DME, and PLACID [13,36].

CI, confidence interval; ETDRS, Early Treatment Diabetic Retinopathy Study; FE, fixed effects; IVT-AFL, intravitreal aflibercept; OR, odds ratio; RE, random effects; RR, relative risk/risk ratio.

### Safety outcomes

There was moderate heterogeneity for comparisons between IVT-AFL 2q8 and laser (based on two studies) for all serious AEs (I^2^ = 55%), all AEs (I^2^ = 55%), all-serious non-ocular AEs (I^2^ = 52%) and all-causes of mortality (I^2^ = 47%); there was high heterogeneity for all non-ocular AEs (I^2^ = 86%). There was moderate heterogeneity for comparisons between IVR 0.5 mg PRN and laser for all serious ocular AEs (I^2^ = 67%; two studies) (Additional file 1: Appendix 8). None of these direct comparisons achieved statistical significance. The analyses were limited by differences in the precise definition for the safety outcomes. These definitions are listed in Additional file 1: Appendix 9. There were no significant differences in safety outcomes between IVT-AFL 2q8 and IVR 0.5 mg PRN in either the MTC (Table 5) or Bucher analyses (data not reported). However, there were few events reported in the studies, which resulted in wide CI intervals (summarized in Additional file 1: Appendix 8).

**Table 5:** **Indirect comparison (MTC analysis) of IVT-AFL 2q8 versus IVR 0.5 mg PRN for 12-month safety outcomes**

**Outcome**	**Studies (n)**	**FE: effect size [95% CrI]**	**RE: effect size [95% CrI]**
All AEs	5 studies (n = 1739)*	RR = 0.79 [0.55–1.10]	RR = 0.88 [0.64–1.15]
		OR = 0.61 [0.29–1.26]	OR = 0.58 [0.18–1.82]
All serious AEs	5 studies (n = 1739)*	RR = 0.76 [0.47–1.26]	RR = 0.82 [0.47–1.42]
		OR = 0.71 [0.39–1.32]	OR = 0.74 [0.31–1.72]
All serious ocular AEs	5 studies (n = 1739)*	RR = 0.28 [0.06–1.24]	RR = 0.30 [0.05–2.49]
		OR = 0.27 [0.05–1.25]	OR = 0.28 [0.05–2.58]
All serious non-ocular AEs	4 studies (n = 1343)**	RR = 0.60 [0.32–1.14]	RR = 0.67 [0.29–1.66]
		OR = 0.53 [0.24–1.17]	OR = 0.53 [0.12–2.11]
All ocular AEs	4 studies (n = 1343)**	RR = 0.75 [0.54–1.05]	RR = 0.85 [0.58–1.25]
		OR = 0.60 [0.32–1.09]	OR = 0.58 [0.16–1.87]
All non-ocular AEs	3 studies (n = 1215)***	RR = 1.09 [0.87–1.40]	RR = 1.03 [0.80–1.56]
		OR = 1.27 [0.65–2.42]	OR = 1.22 [0.23–6.18]
Eye pain	4 studies (n = 1343)**	RR = 0.98 [0.38–2.70]	RR = 0.96 [0.23–3.91]
		OR = 0.97 [0.34–2.94]	OR = 0.95 [0.17–4.75]
Cataract	3 studies (n = 1215)***	RR = 3.93 [0.77–32.74]	RR = 3.83 [0.52–43.72]
		OR = 4.09 [0.76–34.86]	OR = 4.16 [0.49–50.98]
Hypertension	4 studies (n = 1343)**	RR = 0.95 [0.44–2.07]	RR = 0.95 [0.37–2.55]
		OR = 0.95 [0.40–2.22]	OR = 0.94 [0.28–3.14]
All causes of mortality	3 studies (n = 1215)***	RR = 2.90 [0.20–50.4]	RR = 2.76 [0.13–79.02]
		OR = 3.06 [0.18–60.01]	OR = 2.83 [0.11–85.27]

*VIVID-DME, VISTA-DME, RESTORE, REVEAL, and RELATION [13,15,16,30,31].

**VIVID-DME, VISTA-DME, RESTORE, and RELATION [13,15,30,31].

***VIVID-DME, VISTA-DME, and RESTORE [13,15].

AE, adverse event; CrI, credible interval; FE, fixed effects; IVR, intravitreal ranibizumab; IVT-AFL, intravitreal aflibercept; MTC, mixed treatment comparison; OR, odds ratio; PRN, as needed; RE, random effects; RR, relative risk/risk ratio.

Direct analyses showed that there was moderate heterogeneity between IVT-AFL 2q8 and laser for increased intraocular pressure (I^2^ = 73%) and vitreous hemorrhage (I^2^ = 60%), and low heterogeneity for cataract (I^2^ = 38%) (Additional file 1: Appendix 10). None of these direct comparisons achieved statistical significance. Indirect analyses showed that there were no significant differences between IVT-AFL 2q8 and dexamethasone 0.7 mg implants for the outcomes: macular edema, reduced visual acuity, vitreous hemorrhage, eye pain, increased intraocular pressure and cataracts; however, there was a trend toward fewer events with IVT-AFL 2q8 compared with dexamethasone 0.7 mg implants (Table 6).

**Table 6:** **Indirect comparison (Bucher analysis) of IVT-AFL 2q8 versus dexamethasone 0.7 mg implants for 12-month safety outcomes**

**Outcome**	**Studies (n)**	**FE: effect size [95% CI]**	**RE: effect size [95% CI]**
Macular edema	2 studies (n = 657)*	RR = 0.22 [0.03–1.67]	RR = 0.22 [0.03–1.64]
		OR = 0.21 [0.03–1.69]	OR = 0.21 [0.03–1.70]
Reduced visual acuity	3 studies (n = 1123)**	RR = 0.64 [0.24–1.67]	RR = 0.64 [0.17–2.40]
		OR = 0.61 [0.21–1.77]	OR = 0.61 [0.21–1.77]
Vitreous hemorrhage	3 studies (n = 1123)**	RR = 0.30 [0.07–1.39]	RR = 0.18 [0.02–1.65]
		OR = 0.28 [0.06–1.38]	OR =0.16 [0.02–1.54]
Eye pain	3 studies (n = 1123)**	RR = 0.80 [0.29–2.21]	RR = 0.78 [0.27–2.21]
		OR = 0.79 [0.26–2.38]	OR = 0.76 [0.24–2.38]
Increased intraocular pressure	3 studies (n = 1123)**	RR = 0.08 [0.02–0.42]	RR = 0.13 [0.01–1.79]
		OR = 0.07 [0.01–0.37]	OR = 0.11 [0.01–1.54]
Cataract	3 studies (n = 1123)**	RR = 0.42 [0.13–1.39]	RR = 0.43 [0.12–1.63]
		OR = 0.40 [0.11–1.40]	OR = 0.41 [0.10–1.64]

*VIVID-DME, and PLACID [13,36].

**VIVID-DME_,_ VISTA-DME, and PLACID [13,36].

CI, confidence interval; FE, fixed effects; IVT-AFL, intravitreal aflibercept; OR, odds ratio; RE, random effects; RR, relative risk/risk ratio.

## Discussion

The aim of this systematic review was to identify and review studies informing the clinical effectiveness of IVT-AFL 2q8 in relation to other DME treatments, and to prepare where possible indirect comparisons of IVT-AFL 2q8 against other regimens licensed outside the USA at the time the analyses were conducted (i.e., IVR 0.5 mg PRN, dexamethasone 0.7 mg implants or fluocinolone acetate 0.2 μg/day implants). The evidence from these specific comparisons showed a benefit of IVT-AFL 2q8 over IVR 0.5 mg PRN for the improvement of mean change from baseline in BCVA (+4.67 letters before adjustment for baseline visual acuity and +4.12 after adjustment), the primary efficacy endpoint of VIVID-DME/VISTA-DME [13], and that approximately 70% fewer patients showed a loss of ≥ 10 ETDRS letters, an exploratory endpoint. These results were consistent in multiple analyses, including both MTC analyses (which included up to 10 studies and 3060 patients with DME) and in Bucher analyses (four studies of 1611 patients with DME), and remained consistent when one study in Asian patients [16] was included or excluded from the Bucher analysis. There were no significant differences between IVT-AL 2q8 and IVR 0.5 mg PRN in safety outcomes (for each of the safety outcomes where quantitative analysis was possible). However, the analysis was limited by differences in definitions of AEs between studies, and the total number of AEs in studies was low resulting in wide CIs.

The evidence also favored IVT-AFL 2q8 over dexamethasone in an indirect analysis of three studies with up to 1123 DME patients. More patients (approximately twice as many) receiving IVT-AFL 2q8 showed a gain of ≥ 10 ETDRS letters compared with those receiving dexamethasone. There were also fewer patients treated with IVT-AFL 2q8 who experienced increased intraocular pressure compared with dexamethasone 0.7 mg implants. There were an additional five safety outcomes (macular edema, reduced visual acuity, vitreous hemorrhage, eye pain and cataract) that showed a non-significant trend in favor of IVT-AFL 2q8. Dexamethasone was recently approved for the treatment of adults with visual impairment due to DME who are pseudophakic or insufficiently responsive/unsuitable for non-corticosteroid therapy. This is more restrictive than the population included in this review, and no data were identified to analyze this subgroup separately.

To date, four key systematic reviews for DME have included IVT-AFL 2q8 [17-19,39]. Based on an indirect analysis of 15 randomized studies and eight observational studies of anti-VEGF therapies (IVT-AFL, intravitreal bevacizumab [IVB], IVR and pegaptanib) by Ollendorf et al. [17], it was concluded that anti-VEGF therapy is associated with sustained visual improvements and reduced rescue laser, but there was insufficient evidence to distinguish between treatments [17]. However, this comparison was based on a less rigorous analysis (pairwise indirect comparisons) – without testing for bias or heterogeneity. The review by Ford et al. [40] included the DA VINCI study as the only source of data for IVT-AFL; as mentioned, DA VINCI has a different loading dose regimen to VIVID-DME and VISTA-DME, and there are only 221 patients in this study, divided over five treatment groups, compared with 406 in VIVID-DME and 466 in VISTA-DME [11,13]. Although the Cochrane review by Virgili et al. [18] included VIVID-DME and VISTA-DME, it focused on endpoints measured in logMAR rather than the more usual change in BCVA from baseline, which is included in this review.

In addition, our analysis did not pool data using different IVR dosing regimens (such as PRN or quarterly) and did not include data from heterogeneous IVR studies or time points, which has also been undertaken in earlier meta-analyses. The most recent review by Regnier et al. [39] included Bayesian network meta-analyses based on eight randomized controlled studies that evaluated IVR 0.5 mg PRN, IVT-AFL 2q8, laser photocoagulation or sham in 1978 patients, and reported 6- and 12-month outcomes. The IVT-AFL data included were from three studies (DA VINCI, VIVID-DME, and VISTA-DME) [11,13]. This review concluded that both IVR 0.5 mg PRN and IVT-AFL 2q8 were statistically superior to laser monotherapy (OR = 5.50 and OR = 3.45, respectively) and that the treatment effect of IVR was numerically, but not statistically, superior to IVT-AFL (OR = 1.59 [95% CrI 0.61–5.37]). However, the analyses relate to one secondary efficacy outcome (relative risk of ≥ 10 letter gain at 12 months), not the primary efficacy outcome in any pivotal phase III study of IVR or IVT-AFL (mean gain in BCVA at 12 months in VIVID-DME/VISTA-DME), and there was a lack of detail on the rationale for data inclusion and extraction, assessment of bias and statistical methods used for adjustment. The current review includes a broader range of interventions (including dexamethasone), and more than one outcome was analyzed. It also uses a rigorous, unbiased approach using validated methods. In terms of the single outcome included in the Regnier analysis (2014) [39], the current review finds also that differences between treatments are not statistically significant, but that the direction of effect favors IVT-AFL 2q8 (OR = 1.64 [95% CrI 0.97–2.78]). The difference in direction of point estimates between the studies appears largely attributable to the selection of studies and data rather than, for instance, choice of treatment effect measure (OR rather than RR) or any statistical adjustments applied by Regnier et al. [39].

Since we conducted the review and analysis presented here, the Diabetic Retinopathy Clinical Research Network (DRCRnet) has published first year results of the Protocol T study, which directly compared the 12-month outcomes of patients with DME randomized to either IVT-AFL (n = 224), IVR (n = 218) or IVB (n = 218) [20]. Study drugs were administered monthly according to a predefined protocol. The mean difference in BCVA (primary endpoint) at 12 months for IVT-AFL 2 mg versus ranibizumab 0.3 mg was +2.1 letters (*P* = 0.03) overall, and +4.7 letters (*P* = 0.003) in patients with baseline letter score < 69 letters. Prespecified ocular AEs and serious AEs, and Antiplatelet Trialists’ Collaboration-defined arterial thromboembolic events were not significantly different between the three anti-VEGF agents. These findings are comparable with those observed in this analysis where the difference between IVT-AFL and IVR was +4.67 letters (MTC analysis). In the Protocol T study, baseline visual acuity was predictive of outcome [20]. In the current analysis, the difference between IVT-AFL and IVR remained (+4.12 letters; MTC analysis) after adjustment for aggregate differences between studies and treatment arms in baseline visual acuity. While this may be an improvement on making no adjustment, incorporating head-to-head studies or individual patient data could provide further strength to the analysis. It must also be noted that Protocol T included IVR 0.3 mg dose and would, therefore, have been excluded from the current analysis of licensed doses (only IVR 0.5 mg would have been included).

The current review has a number of strengths that are inherent with a meta-analysis (including the use of combined data to increase the statistical power to detect an effect). Systematic reviews of high-quality evidence are also regarded at the higher end of the hierarchy of evidence [41]. This review adhered to international recommendations and guidelines in order to reduce bias in publication selection, including pre-specification of inclusion/exclusion criteria and pre-specification of indirect comparisons of interest. Extensive consideration was also given to clinical and statistical heterogeneity, and appropriate stratification of studies by intervention and posology was applied. Based on this, the network of 10 studies informing the IVT-AFL 2q8 versus IVR 0.5 mg PRN indirect comparison (particularly the four studies in the Bucher analysis) was considered sufficiently similar to employ a fixed-effect model in the analysis.

However, despite efforts to minimize bias and heterogeneity, these analyses do have a number of limitations, which are inherent in modeling approaches that use indirect data comparisons. Many studies had unclear or high risk of bias in at least one domain of the Cochrane risk of bias tool, and type 1/type 2 errors that already exist in published studies may also bias any meta-analysis extrapolating that hypothesis. The most common issue was inadequate masking (data not shown). Patient baseline characteristics differed, such as in the mean or range of BCVA of patients (Table 2), or were often poorly reported, which made it difficult to compare populations between studies. The findings are also based on a small number of studies and should be interpreted with caution. Tests of statistical significance are reported without adjustment for multiplicity of outcomes. This paper also included 12-month data only, which was based on availability at the time of the review. Some studies now have longer-term outcomes available. While the scope of the analysis (limited to licensed agents) ensures that the studies and datasets included are not excessively heterogeneous, there are important studies such as Protocol T and RISE/RIDE outside this scope which would be relevant in any broader assessment of comparative effectiveness of anti-VEGF agents.

It must be noted that the paper reports on selected safety outcomes, which were associated with the feasible networks, and did not compare systemic safety among different doses of DME therapies in detail. A meta-analysis of 11 studies (6596 patients) that compared systemic safety in relation to different regimens (doses and frequencies) of ranibizumab treatment (but for age-related macular degeneration [AMD]) identified a possible relationship associated with monthly versus as-needed dosing for cerebrovascular accidents [42]. Another meta-analysis of 21 studies (9557 patients) that compared systemic safety of anti-VEGF treatment in AMD, DME and retinal vein occlusion found no association between anti-VEGF and increased mortality or vascular events [43]. In our analysis, the selection of feasible networks only may have resulted in under-powering, and the introduction of type 2 errors; however, a complete comparison of systemic safety was out of scope.

## Conclusions

This indirect comparison suggests that IVT-AFL 2q8 after a loading dose of 5 monthly injections improved visual acuity outcomes (‘BCVA mean change from baseline’ and ‘loss ≥ 10 ETDRS letters’) in eyes with center-involved DME to a greater extent than IVR 0.5 mg PRN, though VEGF inhibition with either IVT-AFL or IVR is efficacious and appears safe. IVT-AFL 2q8 is associated with a trend toward fewer ocular AEs compared with dexamethasone 0.7 mg implants. Although the study has a number of strengths, including the adherence to international guidelines for performing indirect analyses, inclusion of prespecified inclusion and exclusion criteria, and comprehensive assessment of clinical and statistical heterogeneity, it does have a number of limitations inherent with indirect analyses, the scope is narrow, and the conclusions must be interpreted with caution. Many studies had unclear or high risk of bias in at least one domain of the Cochrane risk of bias tool, and safety outcomes were limited by differences in definitions of events. The number of events reported across studies was low, and the CIs were wide. There is a need for more studies comparing the relative effects of licensed therapies for DME in order to select the best treatment options for our patients; however, the findings from this indirect analysis are comparable to those published in the Protocol T study, which directly compared IVT-AFL with other anti-VEGF agents in patients with DME.

## Additional file

Additional file 1:
**Appendix 1.** The search strategy for Embase (OvidSP) (1974-23.10.2013). Appendix 2 Additional database searches. Appendix 3 The overall network of all included studies, showing direct comparisons by drug, comparator and dose. Appendix 4 Treatment regimens of included studies. Appendix 5 Summary of the risk of bias. Appendix 6 Direct comparison of IVT-AFL 2q8 (plus sham laser) or dexamethasone 0.7 mg implants (plus laser) versus laser (plus sham injection) for gain ≥ 10 ETDRS letters in key studies. Appendix 7 Indirect comparisons of the effects of IVT-AFL 2q8 versus IVR 0.5 mg PRN on 12-month visual outcomes using MTC adjusted for baseline visual acuity score. Appendix 8 Direct comparison of IVT-AFL 2q8 (plus sham laser) or IVR 0.5 mg PRN (plus sham laser) versus laser (plus sham injection) for safety outcomes in key studies. Appendix 9 Safety outcome definitions. Appendix 10 Direct comparison of IVT-AFL 2q8 (plus sham laser) or dexamethasone 0.7 mg implants (plus laser) versus laser (plus sham laser or implant) for safety outcomes in key studies.
