# Supplementary material for: Systematic review and mixed treatment comparison of intravitreal aflibercept with other therapies for diabetic macular edema (DME)
Source: BMC Ophthalmol. 2015 May 15;15:52. doi: 10.1186/s12886-015-0035-x (PMC4467379; doi:10.1186/s12886-015-0035-x)
Supplement: Additional file 1: — Appendix 1. The search strategy for Embase (OvidSP) (1974-23.10.2013). Appendix 2 Additional database searches. Appendix 3 The overall network of all included studies, showing direct comparisons by drug, comparator and dose. Appendix 4 Treatment regimens of included studies. Appendix 5 Summary of the risk of bias. Appendix 6 Direct comparison of IVT-AFL 2q8 (plus sham laser) or dexamethasone 0.7 mg implants (plus laser) versus laser (plus sham injection) for gain ≥ 10 ETDRS letters in key studies. Appendix 7 Indirect comparisons of the effects of IVT-AFL 2q8 versus IVR 0.5 mg PRN on 12-month visual outcomes using MTC adjusted for baseline visual acuity score. Appendix 8 Direct comparison of IVT-AFL 2q8 (plus sham laser) or IVR 0.5 mg PRN (plus sham laser) versus laser (plus sham injection) for safety outcomes in key studies. Appendix 9 Safety outcome definitions. Appendix 10 Direct comparison of IVT-AFL 2q8 (plus sham laser) or dexamethasone 0.7 mg implants (plus laser) versus laser (plus sham laser or implant) for safety outcomes in key studies. [file 12886_2015_35_MOESM1_ESM.docx]

**Journal:** *BMC Ophthalmology*

**Systematic review and mixed treatment comparison of intravitreal aflibercept with other therapies for diabetic macular edema (DME)**

**Supplemental file**

**Appendix 1 The search strategy for Embase (OvidSP) (1974-23.10.2013).**

| **Facet** | **Line** | **Term** | **Hits** |
| --- | --- | --- | --- |
| **Diabetic macular edema terms** | 1 | exp diabetes mellitus/ | 579,114 |
|  | 2 | Diabet$.ti,ab,ot,hw. | 681,896 |
|  | 3 | (T1dm or t1-DM or IDDM or DMI or DM1 or DM-1 or DM-I or TIDM).ti,ab,ot. | 16,400 |
|  | 4 | (T2dm or DM2 or DM-2 or T2-DM or MODY or DM2 or NIDDM or IIDM or TIIDM).ti,ab,ot. | 21,738 |
|  | 5 | ((juvenile$ or child$ or labil$ or brittle$ or ((earl$ or sudden) adj onset)) adj4 DM).ti,ab,ot. | 591 |
|  | 6 | ((adult$ onset or matur$ or late$ or slow or stable) adj4 DM).ti,ab,ot. | 433 |
|  | 7 | ((insulin-dependen$ or non-insulin dependen$ or noninsulin dependen$) adj4 DM).ti,ab,ot. | 218 |
|  | 8 | ((typ$ 2 or typ$ ii or typ$ two or type2 or typeii) adj3 DM).ti,ot,ab. | 4,579 |
|  | 9 | ((keto$ or nonketo$ or autoimmun$ or auto-immun$) adj4 DM).ti,ab,ot. | 179 |
|  | 10 | ((obes$ or non-obes$ or nonobes$) adj4 DM).ti,ab,ot. | 633 |
|  | 11 | Insulin resistance/ | 73,984 |
|  | 12 | (Insulin$ resist$ adj4 DM).ti,ab,ot. | 214 |
|  | 13 | or/1-12 | 721,677 |
|  | 14 | exp Retina maculopathy/ | 35,532 |
|  | 15 | exp Retina macula oedema/ | 9,946 |
|  | 16 | eye oedema/ | 447 |
|  | 17 | retina macula hemorrhage/ | 630 |
|  | 18 | ((parafovea$ or fovea$ or retina$ or macul$ or eye or eyes) adj2 (edema$ or oedema$ or degenerat$ or swell$ or h?emorrhag$ or microaneur?sm$ or micro-aneur?sm$ or (dilat$ adj2 capillar$))).ti,ot,ab. | 32,853 |
|  | 19 | (CSME or CSMO or CME or CMO).ti,ab,ot. | 7,336 |
|  | 20 | or/14-19 | 56,272 |
|  | 21 | 13 and 20 | 9,453 |
|  | 22 | exp diabetic retinopathy/ or ((diabet$ adj2 maculop$) or (diabet$ adj2 macul$ adj2 edema$) or (diabet$ adj2 macul$ oedema$)).ti,ot,ab. | 28,832 |
|  | 23 | (DME or DMO).ti,ot,ab. | 2,289 |
|  | 24 | 2 and 23 | 683 |
|  | 25 | 22 or 24 | 28,857 |
|  | 26 | 21 or 25 | 31,846 |
| **Randomized controlled studies** | 27 | Random$.tw. or clinical trial$.mp. or exp health care quality/ | 3,217,214 |
|  | 28 | Animal/ | 1,891,296 |
|  | 29 | animal experiment/ | 1,723,174 |
|  | 30 | (rat or rats or mouse or mice or murine or rodent or rodents or hamster or hamsters or pig or pigs or porcine or rabbit or rabbits or animal or animals or dogs or dog or cats or cow or bovine or sheep or ovine or monkey or monkeys).ti,ab,ot,hw. | 5,832,413 |
|  | 31 | or/28-30 | 5,832,413 |
|  | 32 | exp human/ | 15,046,117 |
|  | 33 | human experiment/ | 317,572 |
|  | 34 | or/32-33 | 15,047,558 |
|  | 35 | 31 not (31 and 34) | 4,647,208 |
|  | 36 | 27 not 35 | 3,063,570 |
|  | 37 | **26 and 36** | **7,487** |

Studies filter: Wong SS, Wilczynski NL, Haynes RB. **Developing optimal search strategies for detecting clinically sound treatment studies in EMBASE (best sens).** *J Med Libr Assoc* 2006;**94**:41-7.

**Appendix 2 Additional database searches.**

**Rapid appraisal**

Cochrane Database of Systematic Reviews (up to 2013/10)

Database of Abstracts of Reviews of Effects (up to 2013/07)

International Prospective Register of Systematic Reviews (up to 2013/10/24)

NICE Guidance (up to 2010/10/23)

Guidelines International Network (GIN) (up to 2013/10/23)

NIHR Health Technology Assessment Programme (up to 2013/10/23)

National Guidelines Clearinghouse (up to 2013/10/23)

**Supplementary searches (websites)**

NIH ClinicalTrials.gov (Internet)

MetaRegister of Current Controlled Trials (Internet)

WHO International Clinical Trials Registry Platform (Internet) (up to 2013/10)

**Congress searches**

European Society of Retina Specialists (EURETINA), American Academy of Ophthalmology (AAO), American Diabetes Association (ADA), European Association for the Study of Diabetes (EASD), International Society for Pharmacoeconomics and Outcomes Research (ISPOR), and Association for Research in Vision and Ophthalmology (ARVO) from 2008 to 2013/10. The World Ophthalmology Conference (WOC) (2012) and European Society of Ophthalmology (ESO) (2013) were also searched.

**Appendix 3 The overall network of all included studies, showing direct comparisons by drug, comparator and dose.**

| **Main reference** | **Intervention**  **Comparator** | **Trial name/NCT** | **Follow up (months)** | **Included in primary network** | **Included in indirect comparison** | **Reason for not being included in indirect comparison** |
| --- | --- | --- | --- | --- | --- | --- |
| EUCTR2010-022364-12-DE [1]  NCT01331681 [2]  Clinical study report [3]  Korobelnik, 2014 [4] | IVT-AFL 2q4 + sham laser (n = 136)  IVT-AFL 2q4 then 2q8 + sham laser (n = 135)  Sham + macular laser (n = 133) | VIVID-DME | 12 | Yes | Yes |  |
| Korobelnik, 2014 [4]  NCT01363440 [5]  Clinical study report [6] | IVT-AFL 2q4 + sham laser (n = 156)  IVT-AFL 2q4 then 2q8 + sham laser (n = 154)  Sham + macular laser (n = 156) | VISTA-DME | 12 | Yes | Yes |  |
| Lam, 2009 [7] | IVB 1.25 mg (n = 26)  IVB 2.5 mg (n = 25) | Lam 2009 | 3, 6 | Yes but does not form a closed network, therefore excluded from second network | No | Does not form closed network |
| Lim, 2012 [8] | IVB 1.25 mg + IVTA 2 mg (n = 36)  IVB 1.25 mg (n = 38)  IVTA 2 mg (n = 37) | NCT01342159 | 3, 6, 12 | Yes | No | Not connected to network of interest |
|  |  |  |  |  |  |  |
| Shoeibi, 2013 [9]  Ahmadieh, 2008 [10] | IVB 1.25 mg + IVTA 2 mg (n = 37)  IVB 1.25 mg (n = 41)  Sham (n = 37) | NCT00370422 | 3, 4, 6, 9 | Yes | No | No 12 month outcomes |
| Prager, 2010 [11] | IVB 2.5 mg (n = 15)  IVTA/sham 8 mg (n = 15) | Triastin  NCT00682539 | 6 | Yes, but does not form a closed network, therefore excluded from second network | No | Does not form closed network |
| Andrade, 2010 [12] | IVB 1.25 mg + IVTA 4 mg (n = 9)  IVB 1.25 mg (n = 3)  IVTA 4 mg (n = 6)  Total = 65 randomized | Andrade 2010 | 6 | No. Abstract with insufficient data for analysis (no baseline data and visual acuity reported as ‘lines’) | No | Insufficient data |
| Almeida, 2011 [13]  NCT00997191 [14] | IVB 1.5 mg + laser (n = 21)  IVTA 4 mg + laser (n = 20)  FG macular laser (n = 23) | IBETA  NCT00997191 | 12 | Yes | Yes |  |
| Pappas, 2008 [15] | IVB 1.25 mg (n = 27)  IVTA 4 mg + FG or modified grid laser PC (n = 35) | Pappas 2008 | 6 | Yes | No | No 12 month outcomes |
| Azad, 2012 [16] | IVB 1.25 mg (n = 20)  IVTA 4 mg (n = 20)  mETDRS macular grid laser (n = 20) | Azad 2012 | 3, 6 | Yes | No | No 12 month outcomes |
| Rajendram, 2012 [17] | IVB 1.25 mg (n = 42)  mEDTRS MLT laser (n = 38) | BOLT | 4, 12, 24 | Yes | No | Does not form closed network |
| Chakrabarti, 2008 [18] | IVB 1.25 mg + IVTA 4 mg + focal laser (n = 20)  IVB 1.25 mg + focal laser (n = 20)  IVTA 4 mg + focal laser (n = 20) | Chakrabarti 2008 | NR | No. Insufficient information in poster (unclear reporting of data, units to measure visual acuity not given) | No | Insufficient data |
| Solaiman, 2012 [19] | IVB + macular grid PC (n = 16)  IVB (n = 16) | Solaiman 2012 | Unclear | No. Dose not reported | No | Insufficient data |
| Solaiman, 2010 [20] | IVB 1.25 mg (n = 21)  IVB 1.25 mg + modified grid laser PC (n = 22)  Modified grid laser PC (n = 19) | Solaiman 2010 | 6 | No. Single injection of IVB | No | Not connected to network of interest |
| Soheilian, 2012 [21] | IVB 1.25 mg + sham laser (n = 50)  IVB 1.25 mg + IVTA 2 mg + sham laser (n = 50)  FG macular PC + sham injection (n = 50) | NCT00370669 | 3, 6, 9, 12, 18, 24 | Yes | No | Does not form closed network |
| Faghihi, 2008 [22] | IVB 1.25 mg + IVTA 2 mg (n = 41)  IVB 1.25 mg (n = 42)  ETDRS laser (n = 47) | Faghihi 2008 | 4 | Yes | No | No 12 month outcomes |
| Leitao, 2010 [23] | IVB 1.25 mg + 40 mg/ml subtenonian triamcinolone  IVB 1.25 mg  Total = 24 | Leitao 2010 | 3,6 | No. Insufficient information in abstract (unclear numbers per treatment arm, no baseline) | No | Insufficient data |
| Hurtado, 2012 [24] | IVR 0.5 mg (n = 32)  IVR 0.5 mg + IVTA 2 mg (n = 20)  Macular laser PC (n = 28) | Hurtado 2012 | 3 | No. Unclear quantitative data | No | Insufficient data |
| Prunte, 2013 [25] | IVR 0.5 mg + laser (n = 121)  IVR 0.5 mg extend (n = 128)  IVR 0.5 mg PRN (n = 123) | RETAIN  NCT1171976 | Baseline | No follow-up data | No | Insufficient data |
| Brown, 2013 [26] | IVR 0.3 mg + deferred laser (n = 125)  IVR 0.5 mg + deferred laser (n = 127)  Laser + sham injection (n = 130) | RIDE  NCT00473382 | 24 | Yes. Did not make final network as IVR q4 not PRN. Used sham injection plus laser as comparator. Limited 12-month data available with missing patient data | No | No 12 month outcomes |
| Brown, 2013 [26] | IVR 0.3 mg + deferred laser (n = 125)  IVR 0.5 mg + deferred laser (n = 125)  Laser + sham injection (n = 127) | RISE  NCT00473330 | 24 | Yes. Did not make final network as IVR q4 not PRN. Used sham injection plus laser as comparator. Limited 12-month data available with missing patient data | No | No 12 month outcomes |
| Mitchell, 2011 [27] | IVR 0.5 mg + sham laser (n = 116)  IVR 0.5 mg + laser (n = 118)  Sham injections + laser (n = 111) | RESTORE  NCT00687804 | 12 | Yes | Yes |  |
| Ohji, 2012 [28] | IVR 0.5 mg + sham laser (n = 133)  IVR 0.5 mg + laser (n = 132)  Sham injection + laser (n = 131 | REVEAL/  NCT00989989 | 12 | Yes | Yes |  |
| Ristau, 2013 [29]  Lohmann, 2013 [30] | IVR 0.5 mg + FG laser PC (n = 85)  FG laser PC + sham injections (n = 43) | RELATION | 12 | Yes | Yes |  |
| Elman, 2012 [31] | IVR 0.5 mg + prompt laser (n = 187)  IVR 0.5 mg + deferred laser (n = 188)  Sham injection + prompt laser (n = 293)  IVTA 4 mg + prompt laser (n = 186) | NCT00444600 | 12, 24, 36 | Yes | Yes |  |
| Diabetic Retinopathy Clinical Research Network, 2010 [32] | IVR 0.5 mg + FG/PRP laser (n = 113)  IVTA 4 mg + FG/PRP laser (n = 109)  Sham + FG/PRP laser (n = 123) | NCT00445003 | 3, 12 | Yes | Yes |  |
| Diabetic Retinopathy Clinical Research Network, 2011 [33] |  |  |  |  |  |  |
| Comyn, 2013 [34] | IVR 0.5 mg  mETDRS laser  Total = 36 | NCT01223612  LUCIDATE | 12 | Yes. Assume randomization is 24:12 | Yes |  |
| Cserhati, 2013 [35] | IVR (n = 27)  IVR (fixed/PRN) + navigated laser (n = 15)  IVR (PRN) + navigated laser (n = 34) | Cserhati 2013 | 12 | No. Abstract only (no dose) | No | Insufficient data |
| Gil, 2011 [36] | IVTA 4 mg + sham laser (n = 7)  Pan laser PC + sham injection (n = 7) | NCT00668239 | 3,  6 | Yes, but does not form a closed network, therefore, does not make second network | No | No 12 month outcomes |
| Meza-de Regil, 2004 [37] | Laser + IVTA 4 mg  IVTA 4 mg  Total = 120 | Meza 2004 | 3, 6 | No. Single treatment, no PRN | No | Not connected to network of interest |
| Mohamed, 2006 [38] | IVTA 4 mg + laser (n = 38)  IVTA 4 mg (n = 41) | Mohamed 2006 | 6 | No. Single treatment, no PRN | No | Not connected to network of interest |
| Kim, 2010 [39] | IVTA 4 mg + macular laser PC (n = 37 completed, n = 48 randomized)  IVTA 4 mg IVTA (n = 26 completed, n = 38 randomized) | Kim 2010 | 36 | Yes | No | No 12 month outcomes |
| Aydin, 2009 [40] | IVTA 4 mg + laser (n = 17)  Laser + IVTA 4 mg (n = 13)  IVTA 4 mg (n = 19) | Aydin 2009 | 3, 6 | No. Single treatment, no PRN | No | Not connected to network of interest |
| Mirshahi, 2010 [41] | IVTA 4 mg + PR PC + macular PC (n = 18)  PR PC + macular PC (n = 18) | Mirshahi 2010 | 4, 6, 18 | Yes | No | No 12 month outcomes |
| Lam, 2007 [42] | IVTA 4 mg IVTA + sequential grid laser (n = 36)  Grid laser PC (n = 37)  IVTA 4 mg (n = 38) | Lam 2007 | 4, 6 | Yes | No | No 12 month outcomes |
| Diabetic Retinopathy Clinical Research Network, 2009 [43] | IVTA 1 mg (n = 256)  IVTA 4 mg (n = 254)  FG PC (n = 330) | DRCRN 2009 (IVT)  NCT00367133 | 4, 8, 12, 16, 20, 24, 36 | Yes | No | Not connected to network of interest |
| Bordon, 2006 [44] | IVTA 8 mg (n = 15)  Laser (n = 18) | Bordon 2006 | 12 | Yes, but does not form a closed network | No | Does not form closed network |
| Norlaili, 2011 [45] | Laser (n = 20)  IVTA 4 mg (n = 20) | Norlaili 2011 ISRCTN05040192 | 3 | No. Single treatment, no PRN | No | Not connected to network of interest |
| Ockrim, 2008 [46] | IVTA 4 mg (n = 43)  ETDRS laser (n = 45) | Ockrim 2008 | 4, 8, 12 | Yes | No | Not connected to network of interest |
| Maia, 2009 [47] | IVTA 4 mg + PR PC + macular FG PC  Panretinal PR PC + macular FG PC  Total n = 22 | NCT00443521 | 3, 6, 9, 12 | Yes | Yes |  |
| Saraiva, 2008 [48] | Macular grid PC laser + IVTA 4 mg  IVTA 4 mg  Macular grid PC laser  Total n = 30 | Saraiva 2008 | 6 | No. Number per treatment arm not reported. Outcomes not reported in full for extraction | No | Insufficient data |
| Gillies, 2010 [49] | mETDRS laser + IVTA 4 mg (n = 42)  mETDRS laser + sham injection (n = 42) | NCT00148265 | 6, 24 | Yes | No | No 12 month outcomes |
| Gillies, 2006 [50] | IVTA 4 mg (n = 34)  Saline placebo (n = 35) | TDMO  NCT001675518 | 3, 24 | Yes | No | No 12 month outcomes |
| Gillies, 2010 [51] | IVTA (n = 27)  Sham (n = 25) | Post-hoc analysis | 36 | Yes | No | No 12 month outcomes |
| Diaz-Rohena, 2012 [52] | Pegaptanib q4 (n = 49)  Pegaptanib q6 (n = 23) | PRESERVE | 6 | Yes but does not form a closed network | No | No 12 month outcomes |
|  |  |  |  |  |  |  |
| Sultan, 2011 [53] | Pegaptanib 0.3 mg (n = 133)  Sham (n = 127)  Pegaptanib 0.3 mg + laser (n = 145)  Sham + laser (n = 143)  Pegaptanib 0.3 mg (n = 133)  sham (n = 127) | NCT00605280 | 12, 24 | Yes but does not form a closed network | No | Does not form closed network |
| NCT01100307 [54] | Pegaptanib 0.3 mg  Sham injection  (n = 243) | NCT01100307 | 3, 6, 12 | Yes but does not form a closed network | No | Does not form closed network |
| Pearson, 2011 [55] | FAc 0.59 mg implant (n = 127)  Standard of Care; laser or observation (n = 69) | NCT00502541 | 1.5, 3, 6, 10, 12, 24, 36, 48 | Yes | No | Does not form closed network |
| Campochiaro, 2011 [56] | FAc 0.2 µg/day (n = 376)  FAc 0.5 µg/day (n = 395)  Sham (n = 185) | FAME | 24, 36 | Yes | No | No 12 month outcomes |
| Pearson, 2004 [57] | FAc 2 mg implant (n = 11)  FAc 0.5 mg implant (n = 41)  Standard of Care (n = 28)  Standard of Care may include macular grid laser or observation | Pearson 2004 | 6, 12, 24 | Yes | No | Does not form closed network |
| Callanan, 2013 [58] | Dexamethasone 0.7 mg implant + laser (n = 126)  Laser + sham injection (n = 127) | PLACID | 1, 4, 6, 9, 12 | Yes, but does not form a closed network | Yes |  |
| Williams, 2006 [59] | Dexamethasone 0.7 mg drug delivery system  Dexamethasone 0.35 mg drug delivery system  Observation | Williams 2006 | 3 | Yes, but does not form a closed network. | No | Does not form closed network |
| Haller, 2010 [60] | Dexamethasone 0.7 mg implant (n = 57)  Dexamethasone 0.35 mg implant (n = 57)  Observation (n = 57) | NCT00035906 | 1, 2, 3, 6 | Yes, but does not form a closed network. Unclear if linked to Williams 2006 | No | Does not form closed network |
| Kuppermann, 2003 [61] | Dexamethasone 0.7 mg implant  Dexamethasone 0.35 mg implant  Observation | Kuppermann 2003 | 3 | Yes, but does not form a closed network. Unclear if linked to NCT00035906 | No | Does not form closed network |
| Tewari,1998 [62] | Diode laser 810 nm  Argon laser 514 nm | Tewari 1998 | 3, 6 | Yes but does not form a closed network | No | Does not form closed network |
| Akduman, 1997 [63] | Diode laser 810 nm (n = 85)  Argon green laser 514 nm (n = 86) | Akduman 1997 | 15 | Yes but does not form a closed network | No | Does not form closed network |
| Figueira, 2009 [64] | Micropulse diode laser 810 nm (n = 44)  Argon green laser 514nm (n = 40) | MP1 | 12 | Yes but does not form a closed network | No | Does not form closed network |
| Laursen, 2004 [65] | Subthreshold micropulse diode laser 814 nm (n = 12)  Argon green laser 514 nm (n = 11) | Laursen 2004a | 6 | Yes but does not form a closed network | No | Does not form closed network |
| Laursen, 2004 [66] | Subthreshold micropulse diode laser 810 nm  Argon laser 514 nm | Laursen 2004b | 5 | Yes but does not form a closed network | No | Does not form closed network |
| Grigorian, 2003 [67] | Subthreshold micropulse diode laser (n = 10)  Argon laser (n = 10) | Grigorian 2003 | 4 | Yes but does not form a closed network | No | Does not form closed network |
| Lavinsky, 2011 [68] | ND-SDM 810 nm (n = 39)  HD-SDM 810 nm (n = 42)  mETDRS focal/grid laser 532nm (n = 42) | NCT00552435 | 12 | Yes but does not form a closed network | No | Does not form closed network |
| Vujosevic, 2010 [69] | Micropulse diode laser 810 nm (n = 32)  Modified EDTRS 514 nm laser (n = 30) | Vujosevic 2010 | 12 | Yes but does not form a closed network | No | Does not form closed network |
| Venkatesh, 2011 [70] | Subthreshold micropulse diode laser (n = 23)  Double frequency neodymium YAG laser (n = 23) | Venkatesh 2011 | 6 | Yes but does not form a closed network | No | Does not form closed network |
| Salman, 2011 [71] | Pascal laser (n = 30)  Conventional laser, macular PC (n = 30) | Salman 2011 | 12 | Yes but does not form a closed network | No | Does not form closed network |
| Blankenship, 1979 [72] | Argon laser PC (n = 39)  Untreated (n = 39) | Blankenship 1979 | 12, 24 | Yes but does not form a closed network | No | Does not form closed network |
| Olk, 1986 [73] | Modified grid argon blue/green laser 488,514 nm (n = 82)  Untreated (n = 78) | Olk 1986 | 12, 24 | Yes but does not form a closed network | No | Does not form closed network |
| Karacorlu, 1993 [74] | Argon green laser 514 nm PC (n = 47)  Dye yellow laser 570 nm PC (n = 38) | Karacorlu 1993 | 12 | Yes but does not form a closed network | No | Does not form closed network |
| Casswell, 1990 [75] | Krypton red laser 647 nm (n = 48)  Argon green/blue laser 488/514 nm (n = 43) | Casswell 1990 | 24 | Yes but does not form a closed network | No | Does not form closed network |
| Khairallah, 1996 [76] | Krypton red laser 647 nm (n = 72)  Argon green laser 514 nm (n = 79) | Khairallah 1996 | 12 | Yes but does not form a closed network | No | Does not form closed network |
| Zhou, 2008 [77] | Krypton red laser 647 nm (n = 76)  Argon green laser 514 nm (n = 78) | Zhou 2008 | 3−36 | Yes but does not form a closed network | No | Does not form closed network |
| Olk, 1990 [78] | Krypton red laser 647 nm (n = 109)  Argon green/blue laser 514 nm (n = 116) | Olk 1990 | 12, 24 | Yes but does not form a closed network | No | Does not form closed network |
| Fong, 2006 [79] | ETDRS laser (n = 92)  Mild macular grid laser (n = 96) | Fong 2006 | 12 | Yes but does not form a closed network | No | Does not form closed network |
| Kumar, 2010 [80] | Subthreshold laser (n = 15)  Threshold laser (n = 15) | Kumar 2010 | 4 | Yes but does not form a closed network | No | Does not form closed network |
| Shao, 2013 [81] | Subthreshold laser (n = 27)  Threshold laser (n = 30) | Shao 2013 | 3 | Yes but does not form a closed network | No | Does not form closed network |
| Patz, 1985 [82] | Deferred focal laser photocoagulation (n = 1490)  Immediate focal laser photocoagulation (n = 754) | Patz 1985 | 12 | Yes but does not form a closed network | No | Does not form closed network |

ETDRS, Early Treatment Diabetic Retinopathy Study; FAc, fluocinolone acetonide; FG, focal grid; HD-SDM, high-density subthreshold diode-laser micropulse photocoagulation; IVB, intravitreal bevacizumab; IVR, intravitreal ranibizumab; IVT-AFL, intravitreal aflibercept; IVTA, intravitreal triamcinolone acetonide; mETDRS, modified ETDRS; MLT, macular laser therapy; ND-SDM, normal density subthreshold diode-laser micropulse photocoagulation; NR, not reported; PC, photocoagulation; PR, panretinal; PRN, as-needed; PRP, panretinal photocoagulation.

**Footnote**

As described in Table 1, to identify which studies would inform our analyses, the following were excluded:

- Studies that were connected by one arm only and did not form a closed network, unless they included comparators of interest.
- Studies which formed loops but did not lie along the path between IVT-AFL 2q8 versus IVR 0.5 mg PRN or IVT-AFL 2q8 versus FAc 0.2 μg/day.
- Studies which did not report 12 month outcomes.

**Appendix 4 Treatment regimens of included studies.**

| **Reference** | **Treatment group** | **Detailed regimen** | **PRN retreatment criteria for drug** | **PRN retreatment criteria for laser** |
| --- | --- | --- | --- | --- |
| VIVID-DME  and VISTA-DME [4] | IVT-AFL + sham laser  Fixed 2q4 | IVT-AFL 2 mg fixed given every 4 weeks (2q4) plus sham laser  Assessed for laser retreatment from week 12 and additional treatment from week 24 | Patients evaluated for additional treatment from week 24 if criteria were met (loss ≥ 10 letters) | Patients were assessed for laser retreatment according to the following: thickening of the retina/hard exudates/at or within 500 μm of the centre of the macula, hard exudates within 500 μm of the centre of the macula |
|  | IVT-AFL + sham laser  Fixed 2q8 | IVT-AFL 2 mg every 4 weeks for 5 doses then every 8 wks (2q8) plus sham laser treatment. Sham injections were given every 8 weeks when IVT-AFL was not given  Assessed for laser retreatment from week 12 and additional treatment from week 24 |  |  |
|  | Laser + sham injections | Laser at baseline and sham injections at every visit. Retreatment assessed from week 12 |  |  |
| IBETA  [13] | Laser  Fixed + PRN | Focal/grid photocoagulation | Retreatment was performed at weeks 20 and 40 if central subfield macular thickness > 275 µm | Focal/grid photocoagulation according to ETDRS guidelines |
|  | IVB + laser photocoagulation  Fixed + PRN | IVB 1.5 mg plus focal photocoagulation after 4 weeks |  |  |
|  | IVTA + laser  Fixed + PRN | IVTA 4 mg plus focal photocoagulation after 4 weeks |  |  |
| RESTORE [27] | laser + sham injections  Fixed q4 then PRN | 3 monthly IVR 0.5 mg injections (or sham) at 0−2 months. Further monthly injections according to retreatment criteria.  Laser treatment regimen not reported | From month 3, 1 injection/month was to be continued if stable visual acuity was not reached  Injections could be resumed PRN if there was a decrease in BCVA due to DME progression  Retreatments were given at intervals > 3 months from the previous treatment if deemed necessary | Retreatment was according to ETDRS guidelines  Patients receiving retreatment with active or sham laser continued to be treated with monthly ranibizumab or sham injections as long as the treatment criteria for intravitreal injection were fulfilled |
|  | IVR + sham laser  Fixed q4 then PRN |  |  |  |
|  | IVR + laser  Fixed q4 then PRN |  |  |  |
| REVEAL  [28] | IVR + sham laser  Fixed q4 then PRN | IVR 0.5 mg/sham injections were given on day 1, month 1 and month 2 then PRN | If stable vision not reached at month 3, 1 injection/month continued until stable vision was reached | Active/sham laser photocoagulation was performed according to ETDRS guidelines at ≥ 3-month intervals |
|  | IVR + prompt laser  Fixed q4 then PRN |  |  |  |
|  | Laser + sham injections  Fixed q4 then PRN |  |  |  |
| RELATION [29,30] | IVR + prompt laser  Fixed q4 then PRN | Active laser photocoagulation at baseline  IVR 0.5 mg at baseline, 30, 60 and 90 days, then PRN | Retreatment reapplied at intervals no shorter than 28 days from last treatment | Focal/grid photocoagulation  Retreatment was applied at no shorter than 3 months from the last treatment |
|  | laser + sham injections  Fixed q4 then PRN | Active laser photocoagulation at baseline  Sham intravitreal injections at baseline, 30, 60 and 90 days, then PRN |  |  |
| DRCR.net Protocol I [32] | Prompt laser + sham injections  Fixed q4 then PRN | Laser (1 week after first sham) + sham injections every 4−12 weeks, and PRN from 16 weeks | From week 16, drug was given unless deemed a ‘success’ (visual acuity letter score 84 [20/20] or OCT central subfield thickness < 250 µm since last treatment  From week 24, retreatment was given if the study visit was deemed ‘no improvement’  Retreatment with IVR could be every 4 weeks; IVTA every 16 weeks | Focal/grid photocoagulation administered using modified ETDRS protocol |
|  | IVR + prompt laser  Fixed q4 then PRN | IVR 0.5 mg every 4−12 weeks, and PRN from 16 weeks  Laser given 7−10 days after first injection |  |  |
|  | IVR + deferred laser  Fixed q4 then PRN | IVR 0.5 mg every 4−12 weeks, and PRN from 16 weeks  Laser given 24 days after first injection |  |  |
|  | IVTA + laser  Fixed q4 then PRN | IVTA 4 mg at randomization plus laser 1 week post-injection, repeated every 16 weeks with sham at 4-week intervals, and PRN from 16 weeks |  |  |
| DRCR.net  Protocol J [33] | Laser + sham injections  Fixed then PRN | Two injections (IVR/sham) were given, one at baseline and one at 4 weeks  After the 14-week visit, additional treatments were at the investigator's discretion | At the investigator's discretion | The focal/grid laser technique was modified from the original ETDRS protocol |
|  | IVR + laser  Fixed then PRN |  |  |  |
|  | IVTA + laser  Fixed then PRN | IVTA 4 mg was given at baseline and a sham injection at week 4  After the 14-week visit, additional treatment was at the investigator's discretion |  |  |
| LUCIDATE [34] | IVR  Fixed q4 then PRN | IVR 0.5 mg at baseline, 4 and 8 weeks then 4-weekly as required to 44 weeks | Not reported | Argon laser therapy to the macula in accordance with the modified ETDRS protocol  PRN every 12 weeks if clinically significant macular edema present |
|  | Laser  Fixed then PRN | Argon laser at baseline, 12, 24 and 36 weeks |  |  |
| Maia, 2009 [47] | Laser  Fixed then PRN | After completion of the bilateral PRP session at week 3, one eye (per patient) was randomly assigned to receive one IVTA 4 mg, and fellow eyes received no additional treatment (control group) | Not reported | Laser photocoagulation performed in three episodes (at weeks 1, 2, and 3) according to ETDRS guidelines |
|  | IVTA + laser  Fixed then PRN |  |  |  |
| PLACID  [58] | Dexamethasone + laser  Fixed then PRN | Dexamethasone 0.7 mg at baseline and laser 1 month later | Retreatment was evaluated at months 4, 6, and 9. Patients had to meet all 4 of the following criteria:  Time domain-OCT central subfield thickness ≥ 250 µm  Minimum interval between treatments of at least 3 months for laser and at least 6 months for dexamethasone/sham  Patient not at significant risk from retreatment  Patient may benefit from retreatment | Modified ETDRS focal (direct/grid) laser photocoagulation using yellow- or green-wavelength laser |
|  | Laser + sham injection  Fixed then PRN | Sham injection at baseline and laser 1 month later |  |  |

BCVA, best-corrected visual acuity; DME, diabetic macular edema; ETDRS, Early Treatment Diabetic Retinopathy Study; IVT-AFL, intravitreal aflibercept; IVB, intravitreal bevacizumab; IVR, intravitreal ranibizumab; IVTA, intravitreal triamcinolone acetonide; OCT, optical coherence tomography; PRN, as needed; PRP, panretinal photocoagulation.

**Appendix 5 Summary of the risk of bias.**

| **Study** | **Random sequence generation** | **Allocation concealment** | **Blinding participants/personnel** | **Blinding outcome assessment** | **Incomplete outcome data** | **Selective reporting** | **Other** |
| --- | --- | --- | --- | --- | --- | --- | --- |
| VIVID-DME [4] | L | L | H | L | L | L | H |
| VISTA-DME [4] | L | L | H | L | L | L | H |
| IBETA [13] | U | U | H | U | H | H | H |
| RESTORE [27] | L | U | H | H | L | L | U |
| REVEAL [28] | U | U | U | U | U | L | U |
| RELATION [29,30] | U | U | U | U | L | U | U |
| DRCR.net Protocol I [32] | U | U | U | L | L | L | L |
| DRCR.net Protocol J [33] | U | U | U | L | U | L | H |
| LUCIDATE [34] | U | U | U | U | U | U | U |
| Maia, 2009 [47] | U | U | H | L | H | U | L |
| PLACID [58] | L | L | L | L | L | L | U |

Risk of Bias: L = low; U = unclear; H = high.

**Appendix 6 Direct comparison of IVT-AFL 2q8 (plus sham laser) or dexamethasone 0.7 mg implants (plus laser) versus laser (plus sham injection) for gain ≥ 10 ETDRS letters in key studies. Indirect comparison (IVT-AFL 2q8 vs dexamethasone 0.7 mg implants) (Bucher analysis) also shown. CI, confidence interval; ETDRS, Early Treatment Diabetic Retinopathy Study; IVT-AFL, intravitreal aflibercept; VEGF, vascular endothelial growth factor.** [4,58]


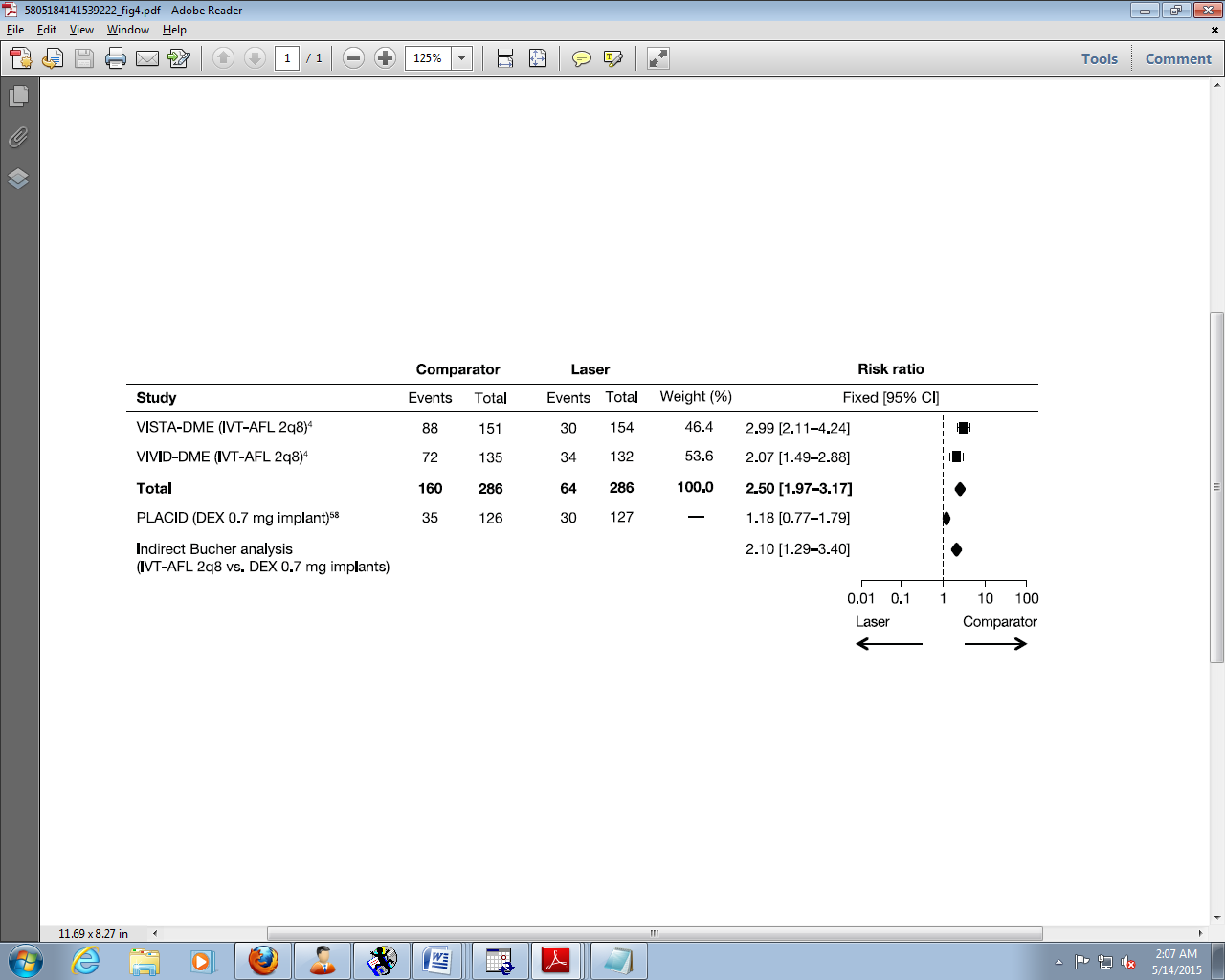


**Appendix 7 Indirect comparisons of the effects of IVT-AFL 2q8 versus IVR 0.5 mg PRN on 12-month visual outcomes using MTC adjusted for baseline visual acuity score.**

| 1. **MTC** | **Studies (n)** | **FE: effect size [95% CrI]** | **RE: effect size [95% CrI]** |
| --- | --- | --- | --- |
| BCVA mean change from baseline | 10 studies (n = 3060)* | MD = 4.12 [1.47-6.81] | MD = 4.15 [0.13-7.94] |
| Gain ≥ 10 ETDRS letters | 6 studies (n = 2810)** | RR = 1.36 [0.97-1.87] | RR = 1.20 [0.81-1.74] |
| Loss ≥ 10 ETDRS letters | 6 studies (n = 2810)** | RR = 0.11 [0.02-0.46] | RR = 0.12 [0.02-0.67] |
| Gain ≥ 15 ETDRS letters | 6 studies (n = 2810)** | RR = 1.45 [0.82-2.50] | RR = 1.30 [0.75-2.30] |
| Loss ≥ 15 ETDRS letters | 6 studies (n = 2810)** | RR = 0.06 [0.00-0.79] | RR = 0.05 [0.00-1.41] |

The adjustment was made in the MTC model by including a treatment interaction effect common across interventions.

* VIVID-DME, VISTA-DME, IBETA, RESTORE, REVEAL, RELATION, DRCR.net Protocol I, DRCR.net Protocol J, LUCIDATE and Maia et al (2009) [4,13,27-30,32-34,47].

** VIVID-DME, VISTA-DME, RESTORE, REVEAL, DRCR.net Protocol I, and DRCR.net Protocol J [4,27,28,32,33].

AFL, aflibercept; CrI, credible interval; FE, fixed effects; IVR, intravitreal ranibizumab; IVT, intravitreal; MD, mean difference; MTC, mixed treatment effect; PRN, as needed; RE, random effects; RR, risk ratio.

**Appendix 8 Direct comparison of IVT-AFL 2q8 (pus sham laser) or IVR 0.5 mg PRN (plus sham laser) versus laser (plus sham injection) for safety outcomes in key studies.**

| **Outcome** | **Studies** | **Events (n)** | **RR [95% CI]** | **I2** | **p-value** |
| --- | --- | --- | --- | --- | --- |
| All AE | 2 studies* | IVT-AFL 2q8 (258) Laser (258) | 1.00 [0.95–1.06] | 55% | 0.98 |
|  | 2 studies** | IVR 0.5 mg PRN (50) Laser (34) | 1.41 [0.95–2.09] | 0% | 0.09 |
| All serious AE | 2 studies* | IVT-AFL 2q8 (72) Laser (78) | 0.93 [0.70–1.22] | 55% | 0.58 |
|  | 2 studies** | IVR 0.5 mg PRN (44) Laser (36) | 1.17 [0.78–1.75] | 0% | 0.44 |
| All serious ocular AE | 2 studies* | IVT-AFL 2q8 (5) Laser (12) | 0.42 [0.15–1.17] | 0% | 0.10 |
|  | 2 studies** | IVR 0.5 mg PRN (8) Laser (4) | 1.81 [0.59–5.62] | 67% | 0.30 |
| All serious non-ocular AE | 2 studies* | IVT-AFL 2q8 (64) Laser (65) | 0.99 [0.73–1.34] | 52% | 0.94 |
|  | 1 study*** | IVR 0.5 mg PRN (23) Laser (15) | 1.47 [0.81–2.66] | NR | NR |
| All ocular AE | 2 studies* | IVT-AFL 2q8 (167) Laser (185) | 0.90 [0.79–1.03] | 0% | 0.12 |
|  | 1 study*** | IVR 0.5 mg PRN (49) Laser (43) | 1.09 [0.80–1.49] | NR | NR |
| All non-ocular AE | 2 studies* | IVT-AFL 2q8 (217) Laser (213) | 1.02 [0.93–1.12] | 86% | 0.68 |
|  | 1 study*** | IVR 0.5 mg PRN (67) Laser (68) | 0.94 [0.76–1.17] | NR | NR |
| Eye pain | 2 studies* | IVT-AFL 2q8 (21) Laser (18) | 1.18 [0.65–2.14] | 0% | 0.59 |
|  | 1 study*** | IVR 0.5 mg PRN (12) Laser (11) | 1.04 [0.48–2.27] | NR | NR |
| Cataract | 2 studies* | IVT-AFL 2q8 (14) Laser (15) | 0.93 [0.46–1.90] | 38% | 0.85 |
|  | 1 study*** | IVR 0.5 mg PRN (2) Laser (7) | 027 [0.06–1.29] | NR | NR |
| Hypertension | 2 studies* | IVT-AFL 2q8 (47) Laser (51) | 0.92 [0.64–1.32] | 0% | 0.67 |
|  | 2 studies** | IVR 0.5 mg PRN (15) Laser (15) | 0.96 [0.48–1.91] | 0% | 0.91 |
| All causes of mortality | 2 studies* | IVT-AFL 2q8 (5) Laser (2) | 2.19 [0.49–9.73] | 47% | 0.30 |
|  | 1 study*** | IVR 0.5 mg PRN (2) Laser (2) | 0.96 [0.14–6.67] | NR | NR |

* VIVID-DME and VISTA-DME; ** RESTORE and REVEAL; *** RESTORE. [4,27,28]

AE, adverse event; CI, confidence interval; IVT-AFL, intravitreal aflibercept; IVR, intravitreal ranibizumab; NR, not reported; PRN, as-needed; RR, risk ratio.

**Appendix 9 Safety outcome definitions.**

| **Outcome/study** | **VIVID-DME**  **[4]** | **VISTA-DME**  **[4]** | **RESTORE**  **[27]** | **REVEAL**  **[28]** | **RELATION**  **[29,30]** |
| --- | --- | --- | --- | --- | --- |
| All serious AE | Any serious TEAE | Any serious TEAE | Total ocular serious AE and total non-ocular serious AE | Serious AE | Total serious AE |
| All serious ocular AE | Any ocular serious TEAE in study eye | Any ocular serious TEAE in study eye | Total ocular serious AE | Serious eye disorders | Serious eye disorders |
| All serious non-ocular AE | Any non-ocular serious TEAE | Any non-ocular serious TEAE | Total non-ocular serious AEs | NR | Total serious AE minus serious eye disorders |
| All AE | Any TEAE | Any TEAE | Total AE potentially related to systemic VEGF inhibition | AE (not including serious) | AE (not including serious) |
| All ocular AE | Any ocular TEAE in study eye | Any ocular TEAE in study eye | Total ocular AE  (at least 3% in any group) | NR | Eye disorders |
| All non-ocular AE | Any non-ocular TEAE | Any non-ocular TEAE | Total non-ocular AE (at least 3% in any group) | NR | NR |

AE, adverse events; NR, not reported; TEAE, treatment emergent adverse events; VEGF, vascular endothelial growth factor.

**Appendix 10 Direct comparison of IVT-AFL 2q8 (pus sham laser) or dexamethasone 0.7 mg implants (plus laser) versus laser (plus sham laser or implant) for safety outcomes in key studies.**

| **Outcome** | **Studies** | **Events (n)** | **RR [95% CI]** | **I^2^** | **P-value** |
| --- | --- | --- | --- | --- | --- |
| Macular edema | 1 study* | IVT-AFL 2q8 (2) Laser (5) | 0.39 [0.08–2.00] | NR | NR |
|  | 1 study** | Dexamethasone 0.7 mg (7) Laser (4) | 1.78 [0.53–5.92] | NR | NR |
| Reduced visual acuity | 2 studies*** | IVT-AFL 2q8 (15) Laser (25) | 0.60 [0.32–1.10] | 0% | 0.10 |
|  | 1 study** | Dexamethasone 0.7 mg (12) Laser (13) | 0.94 [0.45–1.98] | NR | NR |
| Vitreous hemorrhage | 2 studies*** | IVT-AFL 2q8 (4) Laser (11) | 0.36 [0.12–1.13] | 60% | 0.08 |
|  | 1 study** | Dexamethasone 0.7 mg (12) Laser (8) | 1.52 [0.65–3.60] | NR | NR |
| Eye pain | 2 studies*** | IVT-AFL 2q8 (21) Laser (18) | 1.18 [0.65–2.14] | 0% | 0.59 |
|  | 1 study** | Dexamethasone 0.7 mg (13) Laser (9) | 1.47 [0.65–3.31] | NR | NR |
| Increased intraocular pressure | 2 studies*** | IVT-AFL 2q8 (12) Laser (10) | 1.19 [0.53–2.71] | 73% | 0.67 |
|  | 1 study** | Dexamethasone 0.7 mg (25) Laser (2) | 12.70 [3.07–52.49] | NR | NR |
| Cataract | 2 studies*** | IVT-AFL 2q8 (14) Laser (15) | 0.93 [0.46–1.90] | 38% | 0.85 |
|  | 1 study** | Dexamethasone 0.7 mg (13) Laser (6) | 2.20 [0.86–5.61] | NR | NR |

* VIVID-DME; ** PLACID; * VIVID-DME and VISTA-DME. [4,58]

CI, confidence interval; IVT-AFL, intravitreal aflibercept; NR, not reported; RR, risk ratio.

**References**

1. **Clinical trials for eudract_number: 2010-022364-12 (A randomized, double masked, active controlled, phase III study of the efficacy and safety of repeated doses of intravitreal VEGF Trap-Eye in subjects with diabetic macular edema [Bayer HealthCare AG])** [https://www.clinicaltrialsregister.eu/ctr-search/search?query=eudract_number:2010-022364-12].

2. **Intravitreal alfibercept injection in vision impairment due to DME (VIVID-DME). NCT01331681 (Bayer HeathCare AG, Regeneron Pharmaceuticals)** [<http://ClinicalTrials.gov/show/NCT01331681>]

3. Bayer HealthCare AG. **Clinical Study Report: No. PH-37284. BAY 86-5321 / 91745/. 2013;189p.** 2014.

4. Korobelnik JF, Do DV, Schmidt-Erfurth U, Boyer DS, Holz FG, Heier JS, Midena E, Kaiser PK, Terasaki H, Marcus DM, Nguyen QD, Jaffe GJ, Slakter JS, Simader C, Soo Y, Schmelter T, Yancopoulos GD, Stahl N, Vitti R, Berliner AJ, Zeitz O, Metzig C, Brown DM: **Intravitreal aflibercept for diabetic macular edema.** *Ophthalmology* 2014, 08 July [Epub ahead of print].

5. **Study of intravitreal administration of VEGF Trap-Eye (BAY86-5321) in patients with diabetic macular edema. Nct01363440 (Regeneron Pharmaceuticals, Bayer HealthCare AG)** [<http://ClinicalTrials.gov/show/NCT01363440>]

6. **Regeneron Pharmaceuticals. Clinical Study Report: VGFT-OD-1009 (Year 1). 194p.** 2014.

7. Lam DS, Lai TY, Lee VY, Chan CK, Liu DT, Mohamed S, Li CL: **Efficacy of 1.25 MG versus 2.5 MG intravitreal bevacizumab for diabetic macular edema: six-month results of a randomized controlled trial.** *Retina* 2009, **29:**292-299.

8. Lim JW, Lee HK, Shin MC: **Comparison of intravitreal bevacizumab alone or combined with triamcinolone versus triamcinolone in diabetic macular edema: a randomized clinical trial.** *Ophthalmologica* 2012, **227:**100-106.

9. Shoeibi N, Ahmadieh H, Entezari M, Yaseri M: **Intravitreal bevacizumab with or without triamcinolone for refractory diabetic macular edema: long-term results of a clinical trial.** *J Ophthalmic Vis Res* 2013, **8:**99-106.

10. Ahmadieh H, Ramezani A, Shoeibi N, Bijanzadeh B, Tabatabaei A, Azarmina M, Soheilian M, Keshavarzi G, Mohebbi MR: **Intravitreal bevacizumab with or without triamcinolone for refractory diabetic macular edema; a placebo-controlled, randomized clinical trial.** *Graefes Arch Clin Exp Ophthalmol* 2008, **246:**483-489.

11. Prager SG, Kriechbaum K, Mylonas G, Rainer G, Schmidt-Erfurth U, Diabetic Retinopathy Research Group Vienna: **Comparison of intravitreally applied bevacizumab and triamcinolone on diabetic macular edema. Abstract 4262.** Presented at: 2010 Annual Meeting of the Association for Research in Vision and Ophthalmology (ARVO), May 2-6, 2010; Fort Lauderdale, FL.

12. Andrade RE, Oliveira Neto H, Muccioli C, Nobrega MJ, Casella A, Farah ME, Belfort RJ: **A randomized clinical trial to compare the efficacy and safety of isolated or combined intravitreal injection of triamcinolone acetonide and bevacizumab for diabetic macular edema. Abstract 4230.** Presented at: 2010 Annual Meeting of the Association for Research in Vision and Ophthalmology (ARVO), May 2-6, 2010; Fort Lauderdale, FL.

13. Almeida FP, Katayama BY, Messias A, Fisher M, Paccola ML, Costa RA, Scott IU, Jorge R: **Macular laser photocoagulation combined with intravitreal bevacizumab or triamcinolone for diabetic macular edema. Abstract 1300.** Presented at: 2011 Annual Meeting of the Association for Research in Vision and Ophthalmology (ARVO), May 1-5, 2011; Fort Lauderdale, FL.

14. **Intravitreal bevacizumab and triamcinolone associated to laser photocoagulation for diabetic macular edema (IBeTA). NCT00997191 (Fundação de Amparo à Pesquisa do Estado de São Paulo)** [<http://ClinicalTrials.gov/show/NCT00997191>]

15. Pappas GD, Adam CI, Papageorgioy E, Kefalogiannis N, Fanouriakis H: **Triamcinolone and grid laser versus bevacizumab alone for the treatment of diabetic macular edema. E-abstract 3483.** Presented at: IOVS 2008: Annual Meeting of the Association for Research in Vision and Ophthalmology (ARVO), April 27-May 1, 2008; Fort Lauderdale, FL.

16. Azad R, Sain S, Sharma YR, Mahajan D: **Comparison of intravitreal bevacizumab, intravitreal triamcinolone acetonide, and macular grid augmentation in refractory diffuse diabetic macular edema: A prospective, randomized study.** *Oman J Ophthalmol* 2012, **5:**166-170.

17. Rajendram R, Fraser-Bell S, Kaines A, Michaelides M, Hamilton RD, Esposti SD, Peto T, Egan C, Bunce C, Leslie RD, Hykin PG: **A 2-year prospective randomized controlled trial of intravitreal bevacizumab or laser therapy (BOLT) in the management of diabetic macular edema: 24-month data: report 3.** *Arch Ophthalmol* 2012, **130:**972-979.

18. Chakrabarti M, Chakrabarti A, Stephen V, John SR: **Intravitreal monotherapy with bevacizumab and triamcinolone acetonide vs. combination therapy for recalcitrant diabetic macular edema. PO532.** Presented at AAO 2008, November 8-11, 2008; Atlanta, GA*.*

19. Solaiman K, Diab M, Abo-Elenin M: **Repeated intravitreal bevacizumab injection with and without macular grid photocoagulation for treatment for persistent diffuse diabetic macular edema. PO-RET-22.** Presented at: World Ophthalmology Congress (WOC), February 16-20, 2012; Abu Dhabi, UAE.

20. Solaiman KA, Diab MM, Abo-Elenin M: **Intravitreal bevacizumab and/or macular photocoagulation as a primary treatment for diffuse diabetic macular edema.** *Retina* 2010, **30:**1638-1645.

21. Soheilian M, Garfami KH, Ramezani A, Yaseri M, Peyman GA: **Two-year results of a randomized trial of intravitreal bevacizumab alone or combined with triamcinolone versus laser in diabetic macular edema.** *Retina* 2012, **32:**314-321.

22. Faghihi H, Roohipoor R, Mohammadi SF, Hojat-Jalali K, Mirshahi A, Lashay A, Piri N, Faghihi S: **Intravitreal bevacizumab versus combined bevacizumab-triamcinolone versus macular laser photocoagulation in diabetic macular edema.** *Eur J Ophthalmol* 2008, **18:**941-948.

23. Leitao P: **Intravitreal bevacizumab with subtenonian triamcinolone for refractory diabetic macular edema: the functional and anatomic outcome at 6 months.** Presented at: 10th European Society of Retina Specialists (EURETINA) Congress, September 2-5, 2010; Paris, France.

24. Baldivieso Hurtado O, Baldivieso Hurtado R, Baldivieso Hurtado V, Ochoa Contreras D: **Comparison of intravitreal ranibizumab alone or combined with triamcinolone versus macular photocoagulation for treatment of diabetic macular edema. Abstract 397.** Presented at: 2010 Annual Meeting of the Association for Research in Vision and Ophthalmology (ARVO), May, 2-6, 2010; Fort Lauderdale, FL.

25. Prunte C: **Baseline characteristics of the RETAIN study comparing the ranibizumab 0.5mg treatment regimens, treat-and extend vs pro-re-nata in patients with visual impairment die to diabetic macular edema (DME).** Presented at: 13th European Society of Retina Specialists (EURETINA) Congress, September 26-29, 2013; Hamburg, Germany.

26. Brown DM, Nguyen QD, Marcus DM, Boyer DS, Patel S, Feiner L, Schlottmann PG, Rundle AC, Zhang J, Rubio RG, Adamis AP, Ehrlich JS, Hopkins JJ: **Long-term outcomes of ranibizumab therapy for diabetic macular edema: the 36-month results from two phase III trials: RISE and RIDE.** *Ophthalmology* 2013, **120:**2013-2022.

27. Mitchell P, Bandello F, Schmidt-Erfurth U, Lang GE, Massin P, Schlingemann RO, Sutter F, Simader C, Burian G, Gerstner O, Weichselberger A: **The RESTORE study: ranibizumab monotherapy or combined with laser versus laser monotherapy for diabetic macular edema.** *Ophthalmology* 2011, **118:**615-625.

28. Ohji M, Ishibashi T, REVEAL Study Group: **Efficacy and safety of ranibizumab 0.5 mg as monotherapy or adjunctive to laser versus laser monotherapy in Asian patients with visual impairment due to diabetic macular edema: 12-month results of the REVEAL Study. Abstract 4664.** Presented at: 2012 Annual Meeting of the Association for Research in Vision and Ophthalmology (ARVO), May 6-9, 2012.

29. Ristau T, Voegeler J, Lang G, Liakopoulos S, RELATION Study Group: **Relevance of inner versus outer retinal thickness in diabetic macular edema in the RELATION study. Abstract 2376.** Presented at: 2013 Annual Meeting of the Association for Research in Vision and Ophthalmology (ARVO), May 5-9, 2013; Seattle, WA.

30. Lohmann C, Voegeler J, Liakopoulos S, Wiedemann P, Spital G, Lang G, RELATION Study Group: **Double-masked trial demonstrates superiority of combined ranibizumab plus laser versus laser in patients with diabetic macular edema with or without proliferative diabetic retinopathy. Abstract 1239.** Presented at: 2013 Annual Meeting of the Association for Research in Vision and Ophthalmology (ARVO), May 5-9, 2013; Seattle, WA.

31. Elman MJ, Qin H, Aiello LP, Beck RW, Bressler NM, Ferris FL, III, Glassman AR, Maturi RK, Melia M: **Intravitreal ranibizumab for diabetic macular edema with prompt versus deferred laser treatment: three-year randomized trial results.** *Ophthalmology* 2012, **119:**2312-2318.

32. Elman MJ, Aiello LP, Beck RW, Bressler NM, Bressler SB, Edwards AR, Ferris FL, III, Friedman SM, Glassman AR, Miller KM, Scott IU, Stockdale CR, Sun JK: **Randomized trial evaluating ranibizumab plus prompt or deferred laser or triamcinolone plus prompt laser for diabetic macular edema.** *Ophthalmology* 2010, **117:**1064-1077.

33. Googe J, Brucker AJ, Bressler NM, Qin H, Aiello LP, Antoszyk A, Beck RW, Bressler SB, Ferris FL, III, Glassman AR, Marcus D, Stockdale CR: **Randomized trial evaluating short-term effects of intravitreal ranibizumab or triamcinolone acetonide on macular edema after focal/grid laser for diabetic macular edema in eyes also receiving panretinal photocoagulation.** *Retina* 2011, **31:**1009-1027.

34. Comyn O, Peto T, Bunce C, Neveu M, Holder G, Patel P, Egan C, Bainbridge J, Hykin P: **The LUCIDATE study: a randomized clinical trial to evaluate the long-term functional and anatomical effects of repeated ranibizumab therapy compared with laser in diabetic macular edema. Abstract 2390.** Presented at: 2013 Annual Meeting of the Association for Research in Vision and Ophthalmology (ARVO), May 5-9, 2013; Seattle, WA.

35. Cserhati S, Liegl R, Ulbig M, Haritoglou C, Kampik A, Neubauer A, Kernt M: **Combination of ranibizumab and navigated retinal photocoagulation in diabetic macular edema, compared to ranibizumab mono-therapy: twelve month results. Abstract 1240.** Presented at: 2013 Annual Meeting of the Association for Research in Vision and Ophthalmology (ARVO), May 5-9, 2013; Seattle, WA.

36. **Efficacy of macular laser photocoagulation with or without intravitreal injection of bevacizumab (avastin) or triamcinolone acetonide for diffuse diabetic macular edema. IRCT201205029617N1** [[http://www.irct.ir/searchresult.php?id = 9617&number = 1](http://www.irct.ir/searchresult.php?id=9617&number=1)]

37. Meza-de Regil A, Rivera-Sempertegui JO, Martinez-Jardon CS, Leizaola-Fernandez C, Guerrero-Naranjo JL, Quiroz-Mercado H: **Intravitreal triamcinolone acetonide with and without laser grid photocoagulation for the management of persistent diffuse macular edema in diabetics. E-abstract 4095.** Presented at: 2004 Annual Meeting of the Association for Research in Vision and Ophthalmology (ARVO), April 25-29, 2004; Fort Lauderdale, FL.

38. Mohamed S, Chan CK, Lam DSC: **Predictive factors for recurrence of diabetic macular edema. Poster PO372.** Presented at AAO 2006, November 11-14, 2006; Las Vegas, NV.

39. Kim Y, Kang S, Yi CH: **Three-year follow-up of intravitreal triamcinolone acetonide injection and macular laser photocoagulation for diffuse diabetic macular edema. Abstract 4260.** Presented at: 2010 Annual Meeting of the Association for Research in Vision and Ophthalmology (ARVO), May, 2-6, 2010; Fort Lauderdale, FL.

40. Aydin E, Demir HD, Yardim H, Erkorkmaz U: **Efficacy of intravitreal triamcinolone after or concomitant with laser photocoagulation in nonproliferative diabetic retinopathy with macular edema.** *Eur J Ophthalmol* 2009, **19:**630-637.

41. Mirshahi A, Shenazandi H, Lashay A, Faghihi H, Alimahmoudi A, Dianat S: **Intravitreal triamcinolone as an adjunct to standard laser therapy in coexisting high-risk proliferative diabetic retinopathy and clinically significant macular edema.** *Retina* 2010, **30:**254-259.

42. Lam DS, Chan CK, Mohamed S, Lai TY, Lee VY, Liu DT, Li KK, Li PS, Shanmugam MP: **Intravitreal triamcinolone plus sequential grid laser versus triamcinolone or laser alone for treating diabetic macular edema: six-month outcomes.** *Ophthalmology* 2007, **114:**2162-2167.

43. Beck RW, Edwards AR, Aiello LP, Bressler NM, Ferris F, Glassman AR, Hartnett E, Ip MS, Kim JE, Kollman C: **Three-year follow-up of a randomized trial comparing focal/grid photocoagulation and intravitreal triamcinolone for diabetic macular edema.** *Arch Ophthalmol* 2009, **127:**245-251.

44. Bordon AF, Kuczmainski JF, Schirmer M, Lima LSG, Vargas A, Okimoto L: **Photocoagulation versus 8 mg intravitreous trimcinolone acetate (TAAC) for diabetic clinical significant macular edema (CSME): a prospective study. E-abstract 3844.** Presented at: IOVS 2006: Annual Meeting of the Association for Research in Vision and Ophthalmology (ARVO), April 30 - May 4, 2006; Fort Lauderdale, FL.

45. Norlaili M, Bakiah S, Zunaina E: **Intravitreal triamcinolone versus laser photocoagulation as a primary treatment for diabetic macular oedema: a comparative pilot study.** *BMC Ophthalmol* 2011, **11(36)**.

46. Ockrim ZK, Sivaprasad S, Falk S, Roghani S, Bunce C, Gregor Z, Hykin P: **Intravitreal triamcinolone versus laser photocoagulation for persistent diabetic macular oedema.** *Br J Ophthalmol* 2008, **92:**795-799.

47. Maia OO, Jr., Takahashi BS, Costa RA, Scott IU, Takahashi WY: **Combined laser and intravitreal triamcinolone for proliferative diabetic retinopathy and macular edema: one-year results of a randomized clinical trial.** *Am J Ophthalmol* 2009, **147:**291-297.

48. Saraiva FP, Queiroz MS, Costa PG, Gasparin F, Nakashima Y: **[Use of intravitreal triamcinolone and laser photocoagulation for the treatment of diffuse diabetic macular edema].** *Arq Bras Oftalmol* 2008, **71:**493-498.

49. Gillies MC, McAllister IL, Zhu M, Wong W, Louis D, Arnold JJ, Wong TY: **Pretreatment with intravitreal triamcinolone before laser for diabetic macular edema: 6-month results of a randomized, placebo-controlled trial.** *Invest Ophthalmol Vis Sci* 2010, **51:**2322-2328.

50. Gillies MC, Sutter FK, Simpson JM, Larsson J, Ali H, Zhu M: **Intravitreal triamcinolone for refractory diabetic macular edema: two-year results of a double-masked, placebo-controlled, randomized clinical trial.** *Ophthalmology* 2006, **113:**1533-1538.

51. Gillies MC, Islam FM, Larsson J, Pasadhika S, Gaston C, Zhu M, Wong TY: **Triamcinolone-induced cataract in eyes with diabetic macular oedema: 3-year prospective data from a randomized clinical trial.** *Clin Experiment Ophthalmol* 2010, **38:**605-612.

52. Diaz-Rohena R, Kartvelishvili A, Gonzalez VH: **Pegaptanib for Retinal Edema Secondary to Diabetic Vascular Disease (PRESerVe) Study. Abstract 349.** Presented at: 2010 Annual Meeting of the Association for Research in Vision and Ophthalmology (ARVO), May, 2-5, 2010; Fort Lauderdale, FL.

53. Sultan MB, Zhou D, Loftus J, Dombi T, Ice KS: **A phase 2/3, multicenter, randomized, double-masked, 2-year trial of pegaptanib sodium for the treatment of diabetic macular edema.** *Ophthalmology* 2011, **118:**1107-1118.

54. **A phase 3 study to compare the efficacy and safety of 0.3 mg pegaptanib sodium to sham injections in subjects with diabetic macular edema. NCT01100307 (Pfizer)** [<http://ClinicalTrials.gov/show/NCT01100307>]

55. Pearson PA, Comstock TL, Ip M, Callanan D, Morse LS, Ashton P, Levy B, Mann ES, Eliott D: **Fluocinolone acetonide intravitreal implant for diabetic macular edema: a 3-year multicenter, randomized, controlled clinical trial.** *Ophthalmology* 2011, **118:**1580-1587.

56. Campochiaro PA, Brown DM, Pearson A, Ciulla T, Boyer D, Holz FG, Tolentino M, Gupta A, Duarte L, Madreperla S, Gonder J, Kapik B, Billman K, Kane FE: **Long-term benefit of sustained-delivery fluocinolone acetonide vitreous inserts for diabetic macular edema.** *Ophthalmology* 2011, **118:**626-635.

57. Pearson P, Baker C, Eliott D, Ip M, Morse L, Callanan D: **Fluocinolone acetonide intravitreal implant for diabetic macular edema: 2 year results. E-abstract 1111.** Presented at: IOVS 2004: Annual Meeting of the Association for Research in Vision and Ophthalmology (ARVO), April, 25-29, 2004.

58. Callanan DG, Gupta S, Boyer DS, Ciulla TA, Singer MA, Kuppermann BD, Liu CC, Li XY, Hollander DA, Schiffman RM, Whitcup SM: **Dexamethasone intravitreal implant in combination with laser photocoagulation for the treatment of diffuse diabetic macular edema.** *Ophthalmology* 2013, **120:**1843-1851.

59. Williams GA, Haller JA, Kuppermann BD, et al: **Evaluation of an intravitreous dexamethasone drug delivery system in patients with persistent diabetic macular edema. Am Acad Ophthalmol 2006:192.**

60. Haller JA, Kuppermann BD, Blumenkranz MS, Williams GA, Weinberg DV, Chou C, Whitcup SM: **Randomized controlled trial of an intravitreous dexamethasone drug delivery system in patients with diabetic macular edema.** *Arch Ophthalmol* 2010, **128:**289-296.

61. Kuppermann BD, Haller JA, Williams GA, et al: **A randomized controlled trial to assess the safety and efficacy of a dexamethasone implant for diabetic macular edema.** Presented at: AAO 2003, November 15-18, 2003; Anaheim, CA.

62. Tewari HK, Gupta V, Kumar A, Verma L: **Efficacy of diode laser for managing diabetic macular oedema.** *Acta Ophthalmol Scand* 1998, **76:**363-366.

63. Akduman L, Olk RJ: **Diode laser (810 nm) versus argon green (514 nm) modified grid photocoagulation for diffuse diabetic macular edema.** *Ophthalmology* 1997, **104:**1433-1441.

64. Figueira J, Khan J, Nunes S, Sivaprasad S, Rosa A, de Abreu JF, Cunha-Vaz JG, Chong NV: **Prospective randomised controlled trial comparing sub-threshold micropulse diode laser photocoagulation and conventional green laser for clinically significant diabetic macular oedema.** *Br J Ophthalmol* 2009, **93:**1341-1344.

65. Laursen ML, Moeller F, Sander B, Sjoelie AK: **Subthreshold micropulse diode laser treatment in diabetic macular oedema.** *Br J Ophthalmol* 2004, **88:**1173-1179.

66. Laursen ML, Moeller F, Sander B, Sjeolie AK: **Subthreshold micropulse diode laser treatment in diabetic macular edema. A comparative pilot study. E-abstract 4076.** Presented at: IOVS 2004: Annual Meeting of the Association for Research in Vision and Ophthalmology (ARVO), April, 25-29, 2004; Fort Lauderdale, FL.

67. Grigorian RA, Zarbin M, Tutela AC, Bhagat N: **Comparison of subthreshold micropulse diode laser photocoagulation with conventional laser photocoagulation for clinically significant macular edema in diabetic patients. E-abstract 3978.** Presented at: IOVS 2003: Annual Meeting of the Association for Research in Vision and Ophthalmology (ARVO), May 4-9, 2003; Fort Lauderdale, FL.

68. Lavinsky D, Cardillo JA, Melo LA, Jr., Dare A, Farah ME, Belfort R, Jr.: **Randomized clinical trial evaluating mETDRS versus normal or high-density micropulse photocoagulation for diabetic macular edema.** *Invest Ophthalmol Vis Sci* 2011, **52:**4314-4323.

69. Vujosevic S, Bottega E, Casciano M, Pilotto E, Convento E, Midena E: **Microperimetry and fundus autofluorescence in diabetic macular edema: subthreshold micropulse diode laser versus modified early treatment diabetic retinopathy study laser photocoagulation.** *Retina* 2010, **30:**908-916.

70. Venkatesh P, Ramanjulu R, Azad R, Vohra R, Garg S: **Subthreshold micropulse diode laser and double frequency neodymium: YAG laser in treatment of diabetic macular edema: a prospective, randomized study using multifocal electroretinography.** *Photomed Laser Surg* 2011, **29:**727-733.

71. Salman AG: **Pascal laser versus conventional laser for treatment of diabetic retinopathy.** *Saudi J Ophthalmol* 2011, **25:**175-179.

72. Blankenship GW: **Diabetic macular edema and argon laser photocoagulation: a prospective randomized study.** *Ophthalmology* 1979, **86:**69-78.

73. Olk RJ: **Modified grid argon (blue-green) laser photocoagulation for diffuse diabetic macular edema.** *Ophthalmology* 1986, **93:**938-950.

74. Karacorlu S, Burumcek E, Karacorlu M, Arslan O: **Treatment of diabetic macular edema: a comparison between argon and dye lasers.** *Ann Ophthalmol* 1993, **25:**138-141.

75. Casswell AG, Canning CR, Gregor ZJ: **Treatment of diffuse diabetic macular oedema: a comparison between argon and krypton lasers.** *Eye (Lond)* 1990, **4 ( Pt 5):**668-672.

76. Khairallah M, Brahim R, Allagui M, Chachia N: **Comparative effects of argon green and krypton red laser photocoagulation for patients with diabetic exudative maculopathy.** *Br J Ophthalmol* 1996, **80:**319-322.

77. Zhou MW, Wu ZQ, Tan XL, et al: **[Comparison of effects of krypton laser and argon laser in the treatment of diabetic macular edema].** *Int J Ophthalmol* 2008, **8:**758-759.

78. Olk RJ: **Argon green (514 nm) versus krypton red (647 nm) modified grid laser photocoagulation for diffuse diabetic macular edema.** *Ophthalmology* 1990, **97:**1101-1112.

79. Fong DA: **Comparison of modified ETDRS and mild macular grid laser photocoagulation for DME. E-abstract 997.** Presented at: IOVS 2006: Annual Meeting of the Association for Research in Vision and Ophthalmology (ARVO), April 30-May 4, 2006; Fort Lauderdale, FL.

80. Kumar V, Ghosh B, Mehta DK, Goel N: **Functional outcome of subthreshold versus threshold diode laser photocoagulation in diabetic macular oedema.** *Eye (Lond)* 2010, **24:**1459-1465.

81. Shao DP, Ding Y, Liu F, et al: **[Clinical effect of subthreshold photocoagulation for diabetic macular edema using 532 laser].** *Eye Sci* 2013, **13:**1391-1394.

82. Patz A, Rice TA, Murphy RP: **Photocoagulation for diabetic macular edema.** *Arch Ophthalmol* 1985, **103:**1796-1806.
